# Bifurcation phenomena in Taylor–Couette flow in a very short annulus with radial through-flow

**DOI:** 10.1038/s41598-022-26645-6

**Published:** 2022-12-21

**Authors:** Sebastian Altmeyer, M. Sankar, Younghae Do

**Affiliations:** 1 Castelldefels School of Telecom and Aerospace Engineering, Universitat Politècnica de Catalunya, 08034 Barcelona, Spain; 2 Department of General Requirement, University of Technology and Applied Sciences, 516 Ibri, Sultanate of Oman; 3 Department of Mathematics, KNU-Center for Nonlinear Dynamics, Kyungpook National University, Daegu, 41566 Republic of Korea

**Keywords:** Fluid dynamics, Statistical physics, thermodynamics and nonlinear dynamics, Applied mathematics

## Abstract

In this study, the non-linear dynamics of Taylor–Couette flow in a very small-aspect-ratio wide-gap annulus in a counter-rotating regime under the influence of radial through-flow are investigated by solving its full three-dimensional Navier-Stokes equations. Depending on the intensity of the radial flow, either an axisymmetric (pure $m=0$ mode) pulsating flow structure or an axisymmetric axially propagating vortex will appear subcritical, i.e. *below* the centrifugal instability threshold of the circular Couette flow. We show that the propagating vortices can be stably existed in two separate parameter regions, which feature different underlying dynamics. Although in one regime, the flow appears only as a limit cycle solution upon which saddle-node-invariant-circle bifurcation occurs, but in the other regime, it shows more complex dynamics with richer Hopf bifurcation sequences. That is, by presence of incommensurate frequencies, it can be appeared as 1-, 2- and 3-torus solutions, which is known as the Ruelle–Takens–Newhouse route to chaos. Therefore, the observed bifurcation scenario is the Ruelle-Takens-Newhouse route to chaos and the period doubling bifurcation, which exhibit rich and complex dynamics.

## Introduction

Propagating flow structures are common in dynamical systems, and many varieties have been studied experimentally and numerically. For example, the Taylor–Couette flow^1^, one confined to the annulus between two independently driven concentric cylinders, has played a key role in viscosity studies and the refinement of low dynamical system and hydrodynamic instability theories^1–4^. Its geometric simplicity allows for well-controlled experiments, theoretical analyses, and easy verification of numerical simulations.

Apart from the numerous studies on the classic Taylor–Couette flow, modifications have attracted attention. For Newtonian flows, such modifications range from radial temperature dependencies to additional mass flux, either axially through the ends of the annulus or radially through the cylinder walls^5,6^. Other variations include magnetic liquids, such as ferrofluids and magnetohydrodynamic flows. Motivated by applications of dynamic filtration devices^7,8^ the present paper examines the effects of radial mass flux (i.e., injection and suction through the cylinder walls) and the resulting interactions, modifications of flow dynamics, and stability mechanisms. The results may provide new insights to application development^9^, such as rotating filtration systems and flow separation devices, such as for food or oil–sand separation in oil industry^10^.

In a Taylor–Couette system (TCS) with small aspect ratio (e.g., $\Gamma \approx 1$), the flow dynamics is dominated by the competition between “*normal*” and “*anomalous*” flow states, resulting in very rich dynamical behaviors^11–13^. Here the term normal (anomalous) is referred to as a flow state with vortex cells that give an inward (outward) flow near each lid in the radial direction. Thus, for small aspect ratio TCS flow patterns with one (*one-cell flow state*) or two (*two-cell flow state*) Taylor vortex cells were detected^14,15^.

Propagating flow states are observed in TCS, when an inner cylinder and a counter-rotating outer cylinder spin in a viscous liquid. Such flows typically create rotating waves (RWs) that propagate in an azimuthal direction with strong helical contributions^4,16,17^. The most prominent of these rotating structures are classical spiral vortices, which exist either as left- or right-handed types^4,16,18–20^. RWs consist of a pattern that rotates as a whole about the axis of symmetry at a given precession frequency without changing shape, and the frequency is parameter dependent. Thus, RWs describe a 1-torus limit cycle that can be forced by external means (e.g., axial mass flux)^5,21–24^. The appearance of a second frequency increases flow complexity, resulting in a modulated rotating wave solution (MRW)^25,26^, which is next in the hierarchy of solutions (i.e., 2-torus).

Pure axisymmetric propagating solutions, such as propagating vortices (pVs), are unusual and are rarely studied^27–30^. The pV state is characterized by a *toroidal closed* propagating vortex structure that corresponds to a pure axisymmetric $m=0$-mode (see SM for further details regarding the mode spectra) and appears as a primary instability far below corresponding linear stability thresholds^27^. In this study we consider pV states that occur in very short TCSs ($\Gamma =1.3$) with axial stationary end walls under a radial flow. Recently, such axisymmetric pVs have been seen in experiments with ferrofluidic Couette flows with free surface conditions on one side under the influence of an axial homogeneous magnetic field^28^. The observations correspond with those found in classical system setups^27^.

In addition to pVs, we have identified pulsating low states (T2$^\text {puls}$) that are purely axisymmetric but with broken $Z_2$ symmetry. T2$^\text {puls}$ states appear out of a stationary flow structure, T2, which is similar to two previously observed twin-cell flows^31^. Both T2 and twin-cell states consist of two vortex cells dividing the flow in the radial direction. Thus, both touch the top and bottom end walls. The same holds true for the pulsating T2$_{1,2}^\text {puls}$, which appears as a limit cycle and a 2-torus solution. Note that pulsating but axially oscillating flow states have been reported experimentally and numerically^31,32^.

The pV solutions in the present paper differ from those in previous reports as they have a broken mid-height mirror symmetry, $Z_2$ as T2, in contrast to previous ones lacking radial flow and/or having an outer cylinder at rest. These are also complex solutions that incorporate up to three incommensurate frequencies. For classical fluids, all prior-studied pVs were found to be limit cycle solutions^27^ having one characteristic frequency. However, recent works^29,30^ that examined ferrofluids under the influence of versatile (combined) magnetic fields have revealed higher complexities and rich varieties of pV dynamics, some appearing as only quasi-periodic, representing 2-torus solutions. In this study, we identify pVs with one, two, and three incommensurate frequencies, meaning that they live on a limit cycle with 2- and 3-torus invariant manifolds, respectively. Moreover, with the $Z_2$ symmetry broken, all T2 and pV states exist on two symmetry degenerated solution branches.

To summarize, classical TCS studies demonstrate that complex, time-dependent, propagating and/or oscillatory flow patterns can arise when the aspect ratio of the system is reduced^26^. The research gap pertains to the types of dynamical behaviors that can arise in the flow patterns of the TCS when subject to an additional flow, such as a radial one. The features of radial in- and outflow in the present study may also have relevance to the fluid dynamics of astrophysical disks, such as accretions thought to be fed by inflow from external mass sources. A similar combination of motions may occur in the global terrestrial polar vortices, which could may play a role in the transport of ozone in the upper atmosphere^33^.

The current paper is organized as follows. In Section “System setting and numerical procedure”, we clarify the system parameters, describe the numerical method, and define our notation and nomenclature. In Section “Results”, we present our main results in a bifurcation diagram and the corresponding flow structures with a detailed analysis of the flow dynamics in pulsating and propagating flow structures. Finally, we present discussions and conclusions in Section “Discussion and conclusion”.

## System setting and numerical procedure

**Figure 1:**
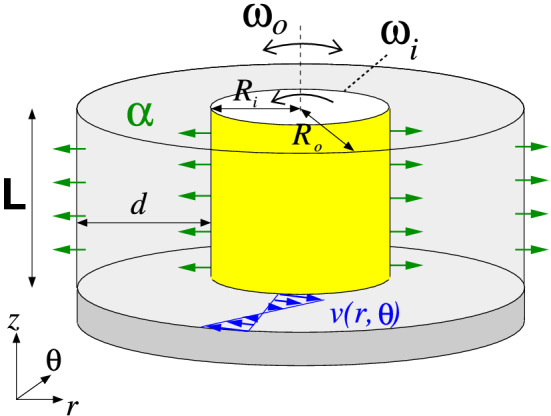
Schematics of the Taylor–Couette system, illustrating radial flow in a counter-rotating configuration, including a sketch of the laminar velocity profile, $v(r,\theta )$ (not to scale). The radial flow can be outward, $(\alpha >0)$ (as shown), or inward, $(\alpha <0)$. Only the bottom lid serving as one of the axial end walls is indicated (dark gray).

Considering the flow in the annular gap between two independently rotating cylinders^1,17^ of length *L*, both are permeable to permit inward or outward radial flow (Fig. 1). The inner cylinder of radius $R_i$ rotates at an angular speed, $\Omega _i$, and the outer cylinder of radius $R_o$ rotates at $\Omega _o$. The end walls at the top and bottom of the annulus are fixed, and the fluid in the annulus is assumed to be Newtonian, isothermal, and incompressible with kinematic viscosity $\nu$. For this work, we assume a wide gap with a fixed radius ratio, $b=0.5$. The length and time scales of the system have a gap width of $d=R_o-R_i$ and a diffusion time of $d^2/\nu$, respectively. The pressure in the fluid is normalized by $\rho \nu ^2 / d^2$. The governing equations are non-dimensional Navier–Stokes:

1 $$\begin{aligned} \partial _t \textbf{u}+(\textbf{u}\cdot \nabla )\textbf{u}= -\nabla p + \nabla ^2 \textbf{u}, \qquad \nabla \cdot \textbf{u}=0, \end{aligned}$$

where $\textbf{u}=(u,v,w)$ is the velocity in cylindrical coordinates, $(r,\theta ,z)$, and the corresponding vorticity is $\nabla \times \textbf{u} = (\xi ,\eta ,\zeta )$. The system is governed by the following independent non-dimensional parameters:

$$\begin{aligned} \text {Inner Reynolds number:}{} \quad & {} Re_i=\Omega _i R_i d/\nu ,\\ \text {Outer Reynolds number:}{} \quad & {} Re_o=\Omega _o R_o d/\nu ,\\ \text {Radial Reynolds number:}{} \quad & {} \mathrm {\alpha }=u_i R_i/\nu \,\, (=u_o R_o/\nu ,\,\, \text {owing to continuity}),\\ \text {Radius ratio:}{} \quad & {} b=R_i/R_o. \\ \text {Aspect ratio:}{} \quad & {} \Gamma =L/d. \end{aligned}$$

Additionally, the ratio of the inner and outer Reynolds numbers is fixed at $Re_i/Re_o=-1$, corresponding to the surface velocities of the two cylinders being equal but opposite, while varying only the radial Reynolds number, $\alpha$, which is positive for a radially outward flow and negative for a radially inward flow. On the cylindrical surfaces, the velocity fields are time independent with $\textbf{u}(r_i,\theta ,z,t)=(u_i,\mathrm {Re_i},0)$ and $\textbf{u}(r_o,\theta ,z,t)=(u_o,\mathrm {Re_o},0)$ with $u_o\,$=$\,b u_i$, respectively, where the non-dimensional inner and outer radii are $r_i= R_i/d=1$ and $r_o= R_o/d=2$ for $b=0.5$. At the top and bottom end walls, the velocity is $u(r,\theta ,-\Gamma /2)=u(r,\theta ,\Gamma /2)=0$. The governing equations and the boundary conditions are invariant under arbitrary rotations, $R_\beta$ (about the axis), the reflection, $K_z$ (about the annulus mid-plan, $z=0$), and the time translations, $\phi _{t_0}$, which generate the symmetry group, $SO(2) \times O(2) \times R$. The first two factors consist of purely spatial symmetries, and the third factor corresponds to the temporal symmetries generating the one-dimensional translation group.

The actions of these symmetries on the velocity field are

2a $$\begin{aligned}&R_\beta (u,v,w)(r,\theta ,z,t) = (u,v,w)(r,\theta +\beta ,z,t), \,\, {\beta \in (0,2\pi ]} \end{aligned}$$

2b $$\begin{aligned}&K_z(u,v,w)(r,\theta ,z,t) = (u,v,-w)(r,\theta ,-z,t), \end{aligned}$$

2c $$\begin{aligned}&\phi _{t_0}(u,v,w)(r,\theta ,z,t) = (u,v,w)(r,\theta ,z,t+t_0), \,\, {t_0\in R}. \end{aligned}$$

For small aspect-ratio regimes, the only axial symmetry in an ideal model is the mid-plane reflection, $Z_2$. Paired with the axisymmetry, *SO*(2), we obtain the complete spatial group of symmetries, $Z_2 \times SO(2)$. Notably, no physical experiment will perfectly fulfill these symmetries, no matter how well-constructed and executed. Owing to unavoidable imperfections, they can only approximately hold, which will results in well-understood modifications to the bifurcation structure^20^.

The Navier–Stokes equations, Eq. (1), are solved using our code G1D3^20^ which is a combination of a finite difference method in the radial and axial directions (*r*, *z*) and a Fourier–Galerkin expansion in the azimuthal direction $(\theta )$ with explicit time splitting, resulting in a decomposition. More details can be found in SM.

### Nomenclature and notation

In this study, we focus on TCS flow dynamics with a small aspect-ratio, $\Gamma =1.3$, motivated by the observation of hysteresis in the range around $\Gamma =1.3$^34^. The counter-rotating cylinders have a fixed outer Reynolds number, $Re_o=-250$, and a fixed inner Reynolds number, $Re_i=250$, where $Re_o/Re_i=-1$. As a result, common structures appearing in the *absence* of any radial flow include RWs and MRWs^26^ having *non-axisymmetric* Fourier modes associated with azimuthal wavenumber(s), $m\ne 0$ (see SM for more details). For the given parameter regime, these are typically $m=\pm 1$ and $m=\pm 2$^1,4,26^. However, in addition to non-axisymmetric states, axisymmetric (pure $m=0$ mode) axial pV flow states have been detected, both stationary (N2, 2-cell flow^26^) and periodic^27^. They display spatiotemporal dynamics with periodic vortex generation and annihilation. Notably, the parameter regime studied here is well-below marginal (linear) stability thresholds. Table 1 indicates the different flow states described in this paper, including acronyms, main characteristics, dominant modes, numbers of vortex cells, flow dynamics, and classifications.

**Table 1:** Flow state nomenclature and abbreviations. From left to right; acronym, flow state, dominant azimuthal (mode) contribution, numbers (#) of vortex cells, flow characteristics, flow dynamics and classification (see also Fig. 3).

Acronym	Flow state	Modes *m*	# vortex cells	Characteristics	Dynamics	Classification
N2	Normal 2-cell	0	2	Stationary	−	Fixed point
pV$_a$	Propagating vortex	0	2 + n n$\in \{0,2\}$	Periodic	Axial propagating	a-torus a $\in \{1,2,3\}$
pV$^u_a$	*Unstable* propagating vortex	0	2 + n n$\in \{0,2\}$	Periodic	Axial propagating	a-torus a $\in \{1,2\}$
T2	Twin pair (4-cells)	0	2 + 2	Stationary	−	Fixed point
T2$_a^\text {puls}$	Pulsating twin pair	0	2 + 2	Periodic	Pulsating	a-torus a $\in \{1,2\}$
RW$_2$	Rotating wave	$0 \gtrdot 2$	2	Periodic	Rotating	1-torus (limit cycle)
RW$_{1,2}$	Rotating wave	$0 \gtrdot 1 \gtrdot 2$	2	Periodic	Rotating	1-torus (limit cycle)
MRW$_{1,2}$	Modulated rotating wave	$0 \gtrdot 1 \gtrdot 2$	2	Periodic	Rotating	2-torus

The relation, $\gtrdot$, indicates that corresponding stimulated (azimuthal) mode amplitudes are larger or smaller. Note all rotating structures exist as *symmetry degenerated* with either dominant positive (*m*) or negative ($-m$) modes (e.g., RW$_2$ and RW$_{-2}$ with dominant $m=2$ or $m=-2$ mode contributions).

## Results

### Bifurcation diagram

**Figure 2:**
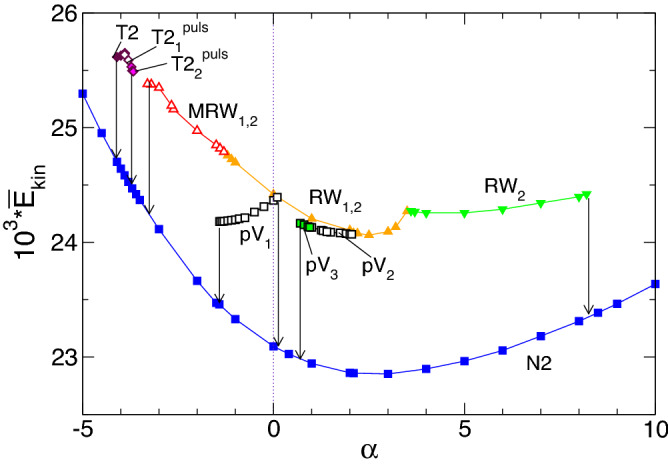
Bifurcation diagram. The total (time-averaged for unsteady flow solutions) modal kinetic energy, $\overline{E}_{kin}$, as a function of the radial Reynolds number, $\alpha$. Different flow structures are labeled, and color-coded symbols are used to identify the flow states. Vertical arrows indicate transition behaviors when a flow state loses stability and changes to another stable state. Solid [open] symbols indicate stationary or rotating time dependent solutions.

**Figure 3:**
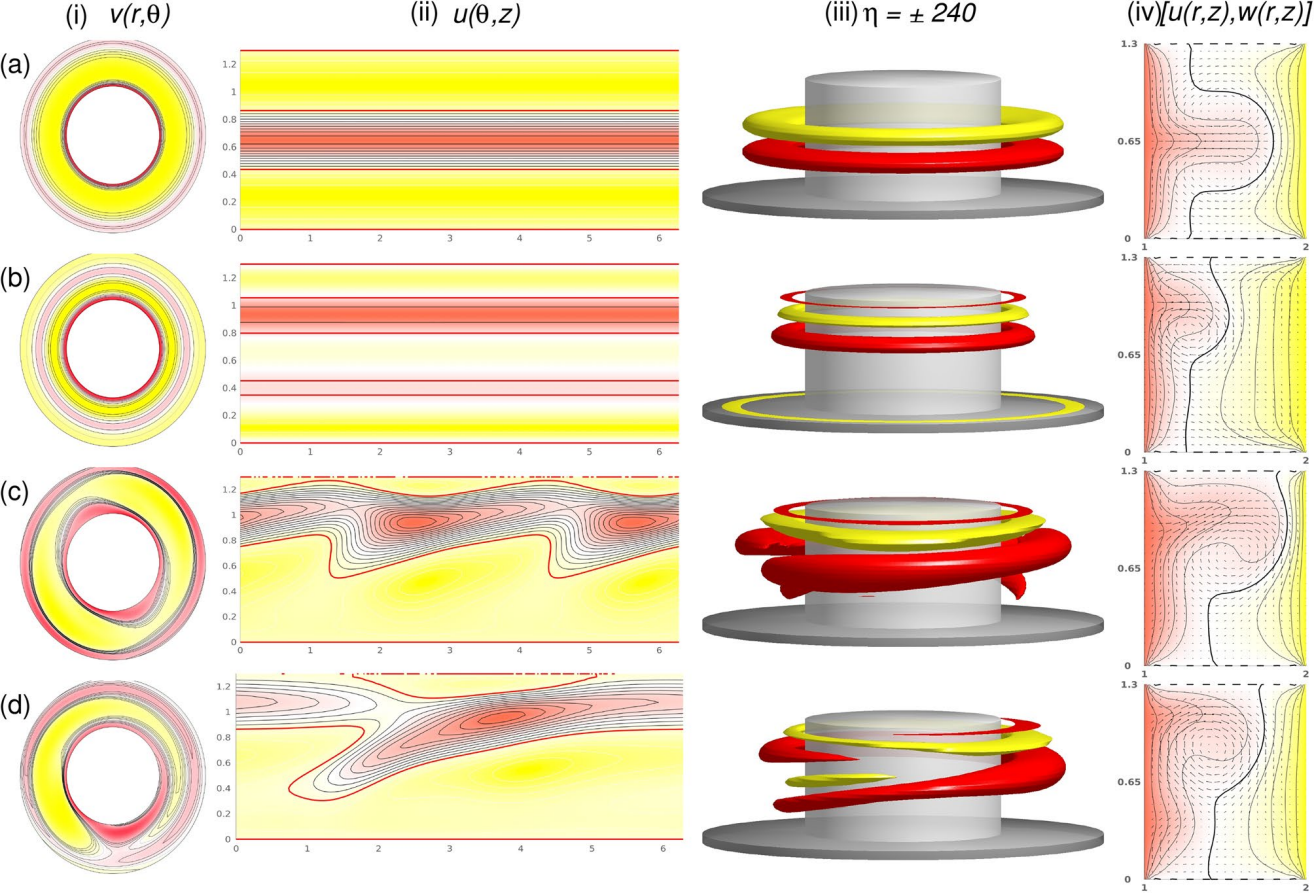
Stationary and rotating flow structures. Shown are (a) N2 at $\alpha =1$; (b) T2 at $\alpha =-4.1$; (c) RW$_2$ at $\alpha =8.2$; and (d) MRW$_{1,2}$ at $\alpha =0$ (see SM Fig. 1 for the corresponding spectra of the mode amplitudes). Shown are (**i**) the azimuthal velocity, $v(\theta ,z)$, at mid-height [red (yellow) color indicates positive (negative) flow, respectively], (**ii**) the radial velocity $u(\theta ,z)$ on an unrolled cylindrical surface in the annulus at mid-gap [red (yellow) color indicates out (in) flow, respectively], (**iii**) isosurfaces of $\eta$ [red (dark gray) and yellow (light gray) colors correspond to positive and negative values, respectively, with zero specified as white] and (**iv**) vector plot [*u*(*r*, *z*), *w*(*r*, *z*)] (at $\theta =0$) of the radial and axial velocity components, including color-coded azimuthal velocity *v* (analog visualizations are used in the following to characterize the flow structures in the paper).

A system bifurcation diagram is presented in terms of the dependence of the variation of time-averaged modal kinetic energy, $\overline{E}_{kin}$, for the different flow states as a function of the applied radial flow, $\alpha$, as shown in Fig. 2. Vertical arrows indicate a transition caused by a change in the stability of a solution. The stationary flow state, N2 (Fig. 3a), is stable over the entire parameter range of $-5\leqslant \alpha \leqslant 10$. N2 has a minimum kinetic energy for a small positive radial (out) flow at $\alpha \approx 2.55$, and its energy increases for both increasing and decreasing $\alpha$, with stronger growth as $\alpha$ becomes more negative (radial inflow).

The pV axisymmetric solution^27^ appears stable in *two separated* $\alpha$ parameter regions. The pV$_1$ solution (1-torus) lies between $-1.411 \leqslant \alpha \leqslant 0.15$, and the kinetic energy, $\overline{E}_{kin}$, continuously decreases with a decreasing $\alpha$. Outside of this range, the pV$_1$ state loses its stability and transitions (vertical arrows) to the stationary flow state, N2. The other region with stable propagating vortices, pV$_2$ and pV$_3$, appears for $0.67\leqslant \alpha \leqslant 2.05$. In this region, an increasing $\alpha$ coincides with a decreasing $\overline{E}_{kin}$. Unlike the first region, pV$_1$, where a 1-torus solution appears, here, the pV states appear as both 2-torus (pV$_2$) and 3-torus (pV$_3$) solutions, and the bifurcation scenario is more complex. Starting at the right edge of this region, a pV$_2$ (2-torus) state appears at $\alpha \approx 2.07$. With a decreasing $\alpha$, pV$_2$ first undergoes a period-doubling (PD) bifurcation at $\alpha \approx 1.47$. As $\alpha$ continues to decrease, a third incommensurate frequency (about 1/3 of the original one) appears at $\alpha \approx 0.98$, resulting in a 3-torus solution, pV$_3$. As $\alpha$ decreases below 0.67, pV$_3$ loses stability, and the flow transitions to N2, which is analogous to the scenario in the pV$_1$ region. When $\alpha$ increases above 2.07, the pV$_2$ state does not fall back to the static N2 state; it instead transitions to the rotating flow state, RW$_{1,2}$. We explain its connection to the stationary flow via unstable pV solution in the next section.

Apart from the different pV states, other RW solutions appear over a wide range of $\alpha$. These RWs have a more complex space-time symmetry as specific combination of rotation and time translation with a characteristic precession period T$_p$, Eq. (3). In the present example, at $\alpha =0$, the RW$_{1,2}$ state occurs, having major ($m=1$) and minor ($m=2$) contributions that remain stable with an increasing $\alpha$ (radial outflow) until $\alpha \approx 3.45$. Here, the $m=1$ mode contribution disappears, and RW$_{1,2}$ changes to a pure rotating wave, RW$_2$, with only a $m=2$ mode contribution. Eventually RW$_2$ loses stability at $\alpha \approx 8.22$ and transitions to the stationary state, N2. Starting at RW$_{1,2}$ at $\alpha =0$ and decreasing $\alpha$ (radial outflow), the flow becomes time-dependent as it undergoes a supercritical Hopf bifurcation at $\alpha \approx -1.23$, which results in a modulated rotating wave, MRW$_{1,2}$, with major ($m=1$) and minor ($m=2$) contributions. As $\alpha$ continues to decrease, MRW$_{1,2}$ loses stability and transitions again to the stationary state, N2.

At even greater inflow (more negative $\alpha$), an isolated island occurs for the parameter regime, $-4.11\leqslant \alpha \leqslant -3.67$, in which another axisymmetric flow solution, mainly consisting of four vortices (T2 - twin pair (4-cell) state^31,32,35^ (Fig. 3(2)) appears. Other than N2, the T2 solution is *not* reflection symmetric about mid-height, indicating a broken $Z_2$ symmetry. For $-4.11\leqslant \alpha \leqslant -3.87$, T2 is stationary but becomes time-dependent (periodic pulsating) with increasing $\alpha$ above $-3.87$, resulting in a limit cycle (1-torus) solution, T2$_1^\text {puls}$. As the magnitude of $\alpha$ continues to decrease, T2$_1^\text {puls}$ first undergoes a PD bifurcation at $\alpha \approx -3.78$ before a second incommensurate frequency (about 1/4 of the original one) is introduced at $\alpha \approx -3.73$, resulting in a 2-torus solution, T2$_2^\text {puls}$. Eventually at $\alpha \approx -3.67$, T2$_2^\text {puls}$ loses its stability, and the flow reverts to the N2 state.

### Flow structures

It is evident from Fig. 2 that a wide range of flow structures can occur in this simple geometry. Here, we describe these flow structures and their characteristics.

#### Stationary and rotating flow structures

Figure 3 presents flow visualizations of different stationary solutions and rotating flow structures corresponding to the flow states in Fig. 2. It is important to note that for all flow structures in this paper, the rotational axisymmetric ($m=0$ mode) contribution is dominant (the best visible in radial velocity $u(\theta ,z)$, e.g. Figs. 3ii and 5(2)). N2 and T2 are *purely* axisymmetric, only containing $m=0$ modes. Special focus should be given to T2, as different time-dependent flow states bifurcate with variations in $\alpha$, which is discussed in detail next.

The N2 solution (2-cell state), which exists over the entire parameter range of $\alpha$ as shown in Fig. 2, represents a stationary, axisymmetric and reflection symmetric (at mid-height $Z_2$) flow, consisting of two large meridional cells^13,26^. Thus, it belongs to the symmetry group, $SO(2) \times O(2) \times R$ [Eq. (2)]. N2 consists of only two large counter-rotating vortices having inflow at the end walls caused by Ekman pumping^36^. This forms an outward jet at mid-height directed from the inner layer towards the outer cylinder boundary layer. In contrast, the presence of a secondary vortex pair near the outer cylinder for the twin-pair solution, T2, results in the compression of the inner vortices closer to the inner cylinder (Fig. 3b,iv). The result is a 4-cell flow structure. For T2, the mid-height reflection-symmetry is broken. However, T2 exists degenerated together with T2$^*$, obtained by simple reflection at mid-height due to operation T2$^*=K_z$T2 (not shown).

Rotating RW and MRW waves exist over a wide range of $\alpha$ (Fig. 2), but their mode contributions change. In particular, the RW structure changes from a pure $m=2$ mode for RW$_2$ (Fig. 3c) to a combination and superposition of $m=1$ and $m=2$ modes for RW$_{1,2}$ (Fig. 3(4)). Flow state RW$_2$ clearly illustrates the $m=2$ contribution, whereas for RW$_{1,2}$ (at $\alpha =0$), $m=1$ dominance (Fig. 3c,ii) is evident in Fig. 3d,ii. Interestingly, we could not detect a stable pure $m=1$ flow state for RW$_1$ like that of the pure RW$_2$.

RWs break system symmetry [Eq. (2)]. RW$_2$ (with azimuthal wavenumber $m=2$) progrades in precision with the rotating inner cylinder. Thus, for RW$_2$, the continuous time translation, $\Phi _\tau$, is replaced with a discrete time translation invariance, $\Phi _{T_P}$, where $T_P$ is the precision period. Classical axisymmetry *SO*(2) is also broken. Generally, for azimuthal wavenumber *m*, the flow is invariant to a cyclic group, $C_m$, as generated by the discrete rotation, $R_{2\pi /m}$. For instance, for RW$_2$ with $m=2$, the axisymmetry, *SO*(2), is replaced by $C_2$ (isomorphic to $Z_2$ group). Regarding the rotating waves, time translations are equivalent to rotations with

3 $$\begin{aligned} \phi _{\tau }(u,v,w)(r,\theta ,z,t) = R_{2\pi \tau /T_P}(u,v,w)(r,\theta ,z,t), \quad \tau \in \mathbb {R}. \end{aligned}$$

The RW$_2$ illustrated in Fig. 3c has a jet emerging from the inner cylinder boundary layer near the upper lit. Applying the $K_z$ reflection to RW$_2$ results in another rotating wave solution state, RW$^*_2=K_z$RW$_2$, with a jet emerging near the bottom lit (here not shown).

**Figure 4:**
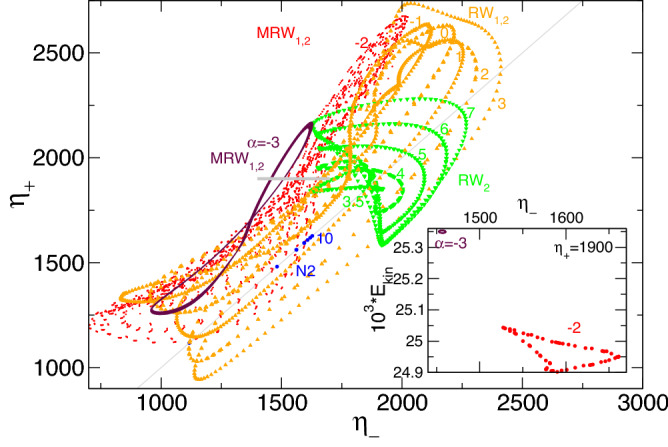
Phase space for rotating flow states. Phase portraits of RW and MRW rotating flow states for $\alpha$, as indicated on $(\eta _-,\eta _+)$. The inset shows the corresponding two-dimensional Poincaré section, $(E_{kin},\eta _-)$, for $\eta _+=1900$ (horizontal gray line in $(\eta _-,\eta _+)$). Numbers in the inset identify corresponding strengths of radial flow $\alpha$.

Although RWs do not necessarily have time dependence apart from their precession time, they illustrate more complex dynamics because of their rotation around the cylinder axis. Figure 4 shows the corresponding phase portrait of $(\eta _-,\eta _+)$. For comparison, N2 states with Z$_2$ symmetry are included (points on the line $\eta _-=\eta _+$) in the Fig. 4 for $\alpha \in \{0,10\}$. With an increasing $\alpha$, the N2 states move in the phase space first up the diagonal line, $\eta _-=\eta _+$ (from bottom left to right in Fig. 4), reaching maximum at radial flow $\alpha \approx 10$, and thereafter moving downwards (from right to left in Fig. 4) with a continually increasing $\alpha$. The distance from the phase portraits to the diagonal line, $\eta _-=\eta _+$, is a measure of the degree to which $Z_2$ symmetry is broken. A corresponding phase portrait of the symmetry-degenerated solutions, RW$^*_2$ and MRW$^*_{1,2}$, can be obtained by exchanging $\eta _-$ and $\eta _+$ (not shown). The inset in Fig. 4 shows the two-dimensional Poincaré section, $(E_{kin},\eta _-)$, for $\eta _+=1,900$ and uncovers the nature of MRW$_{1,2}$ to be as a 2-torus solution.

#### Pulsating flow structures

**Figure 5:**
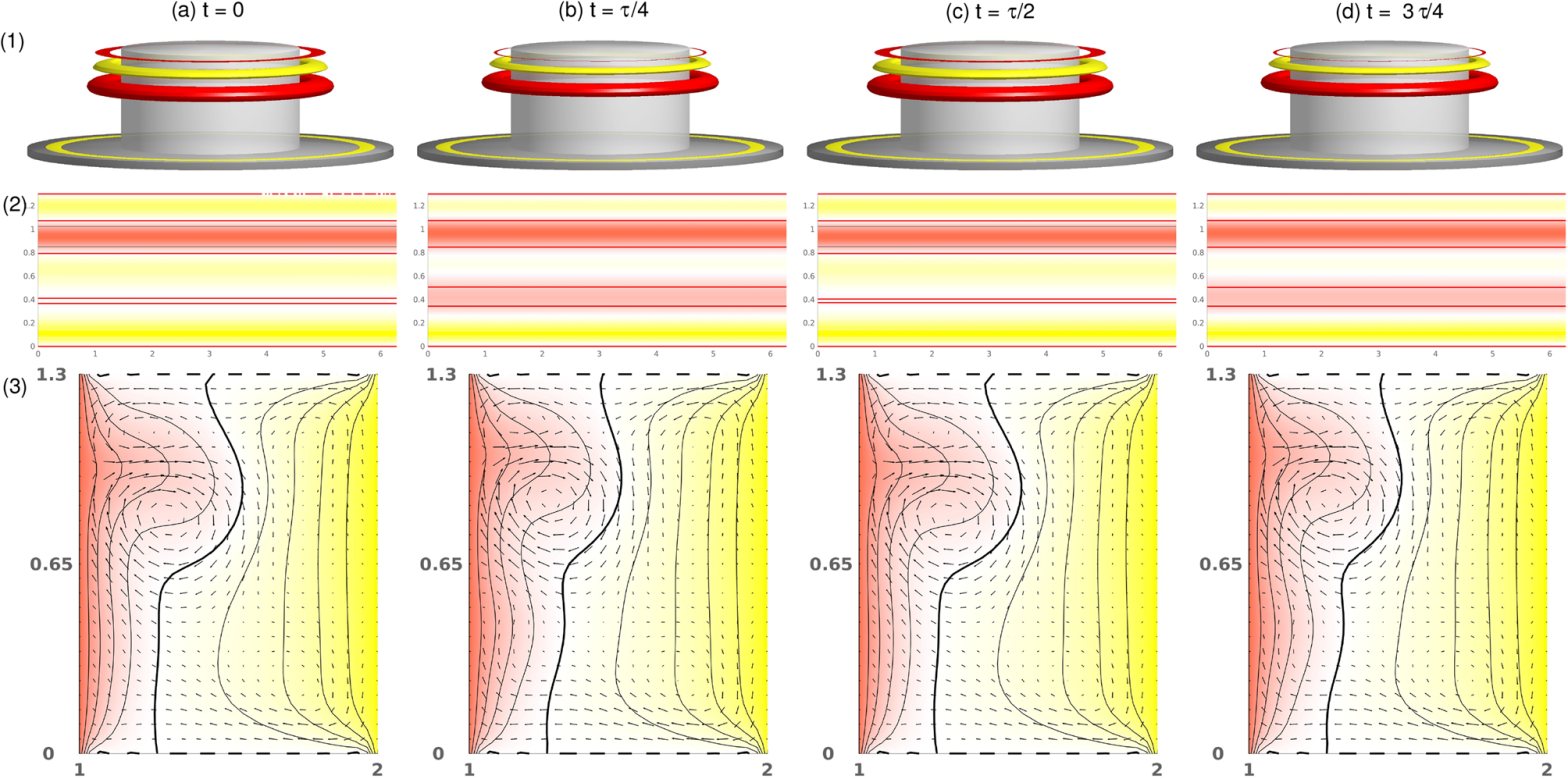
Visualization of the pulsating flow state, T2$_1^\text {puls}$, for $\alpha =-3.7$. One period, $\tau$, at instants of time, *t*, is shown as indicated: (1) isosurfaces of $\eta$ (isolevel shown at $\eta =\pm 240$); (2) Azimuthal velocity at cylinder mid-height. Red (dark gray) and yellow (light gray) colors correspond to positive and negative values, respectively, with zero specified as white. (3) Vector plots [*u*(*r*, *z*), *w*(*r*, *z*)] of the radial and axial velocity component, $(\theta =0)$, where the color-coded azimuthal velocity field, *v*, is shown. The pulsating time is $\tau ^\text {puls}\approx 0.194$. See also the movie, movie_T2_alpha-3_7.avi, in the Supplementary Materials (SMs). The same legends for flow visualization are used for all subsequent visualizations of time dependent flows.

**Figure 6:**
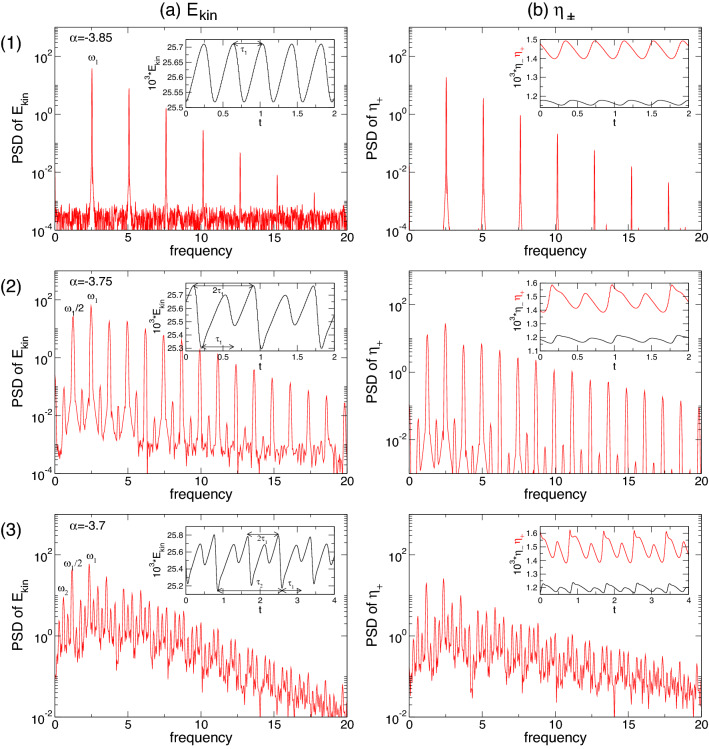
Time series and power spectral densities (PSDs) of the pulsating flow twin-pair state, T2$^{\text {puls}}$, for different $\alpha$. PSDs of (**a**) $E_{kin}$ and (**b**) $\eta _+$ for: (1) T2$_1^{\text {puls}}$ at $\alpha =-3.85$ with pulsating time $\tau _1\approx 0.197$ with corresponding frequency $\omega _1\approx 2.532$; (2) T2$_1^{\text {puls}}$ at $\alpha =-3.75$ (after PD) with pulsating time $2\tau _1\approx 0.407$ with corresponding frequency $\omega _1/2\approx 1.223$; (3) T2$_2^{\text {puls}}$ at $\alpha =-3.7$ with pulsating time $\tau _2\approx 0.4012$ with corresponding frequency $\omega _2\approx 0.618$. Insets show time series of $E_{kin}$, $\eta _+$ (red), $\eta _-$ (black).

When $\alpha$ increases above $-3.74$, the stationary state, T2, loses stability via a supercritical symmetry breaking Hopf bifurcation (discrete time translation). The bifurcating limit cycle solution, T2$_1^{\text {puls}}$ ($\alpha \approx -3.74$), *remains* axisymmetric. The physical manifestation of this new time-dependence is evident as the outward directed jet from the inner cylinder (localized in the basic state) becomes modulated (pulsating). This modulation results from the formation of a second weaker jet emanating from the inner cylinder in the lower part of the bulk (Fig. 5). From its point of emission in the lower part, this secondary jet is directed upwards and eventually merges with the dominant jet in the upper part, resulting in the pulsating characteristics of the flow. See also movie_T2_alpha-3_7.avi in SMs. To gain more insight into the flow dynamics of the pulsating solution, Fig. 5 presents five snapshots of T2$_1^{\text {puls}}$ at $\alpha =-3.7$ over one period, $\tau \approx 0.194$. See also SM, movie_T2_alpha-3_7.avi. Shown are the azimuthal vorticity, $\eta =\pm 240$, the radial velocity, $u(r=0.5d,\theta ,z)$, on an unrolled cylindrical surface, and the vertical cross-section plots of $v[u(r,\theta =0,z),w(r,\theta =0,z)]$, illustrating the pulsation and axial oscillation of the vortex cells. Topologically, the pulsating flow state, T2$_1^{\text {puls}}$, is a limit cycle solution with a frequency of $\omega \approx 2.532$ (see the power spectral densities (PSDs) in Fig. 6a).

The emergence of secondary jets and the ensuing dynamical complexity can be interpreted as a competition between different axial length scales being preferred for the centrifugal instability of the inner cylinder boundary layer as $\alpha$ changes. The short finite system with its axial end walls forces such dynamics and is supported by the fact that for corresponding periodic boundary conditions, no pulsating state is observed. Qualitatively similar states of axially oscillating flow were first detected by Buzug et al.^31^ for classical TCSs and by Altmeyer et al.^35^ for ferrofluidic Couette flows under the influence of external applied magnetic fields.

Figure 6 shows the PSDs of $E_{kin}$ and $\eta _+$ for twin-pair states T2$_1^\text {puls}$ and T2$_2^\text {puls}$ at three different $\alpha$, as indicated. Note, that the time series and PSDs for both global quantity $E_{kin}$ and local quantity $\eta _+$ are presented as $E_{kin}$ may include any hidden symmetries. The insets illustrate corresponding time series of $E_{kin}$ and $\eta _\pm$, including different characteristic times, $\tau _i, \,i\in \{1,2\}$. First, the T2 solution undergoes a supercritical symmetry breaking (time translation) Hopf bifurcation at $\alpha \approx -3.87$, resulting in a time-dependent limit cycle (1-torus) solution, T2$_1^{\text {puls}}$. Close to the onset, the PSD for T2$_1^{\text {puls}}$ at $\alpha =-3.85$ [Fig. 6(1)] indicates a single frequency $\omega _1\approx 2.532$, which corresponds to the pulsating period, $\tau _1\approx 0.197$. With an increasing radial flow, $\alpha$ T2$_l^\text {puls}$ undergoes a PD bifurcation at $\alpha \approx -3.78$. The newly appearing frequency, $\omega _1/2\approx 1.223$, with its corresponding doubled period, $2\tau _1\approx 0.407$, is clearly visible in the PSD for T2$_1^\text {puls}$ at $\alpha =-3.75$ [Fig. 6(2)]. The introduction of a second incommensurate frequency, $\omega _2$ (about 1/4 of the original $\omega _1$), at $\alpha \approx -3.73$ results in the appearance of the 2-torus solution, T2$_2^\text {puls}$. The corresponding PSD becomes significantly more complex owing to various additional frequencies resulting from non-linear interactions and couplings [Fig. 6(3)].

**Figure 7:**
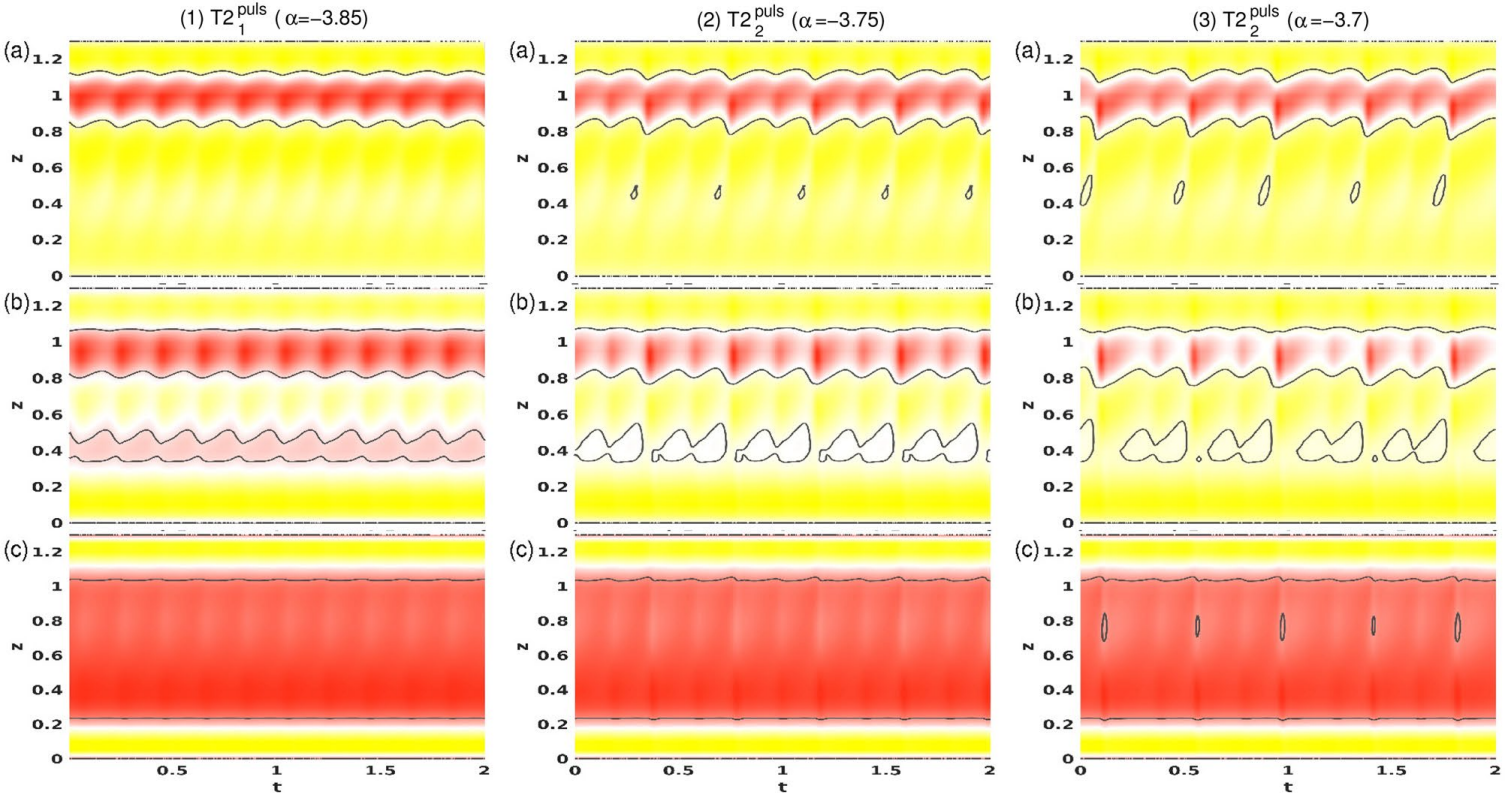
Spacetime plot of the radial velocity *u* for T2$_1^\text {puls}$ at (**1**) $\alpha =-3.85$, (**2**) $\alpha =-3.75$, and T2$_2^\text {puls}$ at (**3**) $\alpha =-3.7.$. Visualizations are shown for three different radial positions, (a) $r=r_1+d/4$, (b) $r=r_1+d/2$, and (c) $r=r_1+3d/4$. Red (dark gray) and yellow (light gray) correspond to positive and negative values, respectively.

Figure 7 provides another perspective of the pulsating flow states, T2$_1^\text {puls}$ and T2$_p^\text {puls}$, at $\alpha$, as indicated (see Fig. 6). Shown are spacetime diagrams of the radial velocity, *u*, at three different radial positions, $u(r=r_1+[d/4,d/2,3d/4],0,z,t)$. The axial asymmetry with the vortex pair placed in the upper half of the bulk is obvious. The zero contour levels are in black and indicate the location where the vortex streams meet in the upper half of the system ($z\approx 0.8$) to separate into the outward directed jets. For $\alpha =-3.85$ the key flow dynamics appear to be dominant in the inner half of the bulk, leaving the outer region nearly unaffected. The footprints of the characteristic modulation/pulsation with the new secondary jet are evident at the mid-gap of the bulk in Fig. 7(1b). The spacetime diagram of T2$_1^\text {puls}$ at $\alpha =-3.75$, following the periodic doubling bifurcation, clearly shows the doubled period, $\tau ^\text {puls}$. Additionally, the flow complexity generally increases, particularly in the interior and center regions of the bulk. Simultaneously, the exterior region becomes more affected. Additional circular regions of zero *u* (Fig. 7(2a)) indicate nascent secondary separations. The variations in the sizes of these circular regions [Fig. 7(3a,3c)] highlight the additional incommensurate frequency of the 2-torus solution, T2$_2^\text {puls}$ (see PSD of T2$_2^\text {puls}$ in Fig. 6(3)). The new frequency is very nearly 1/4 the primary frequency, meaning that it is nearly almost 4 four times the original period.

**Figure 8:**
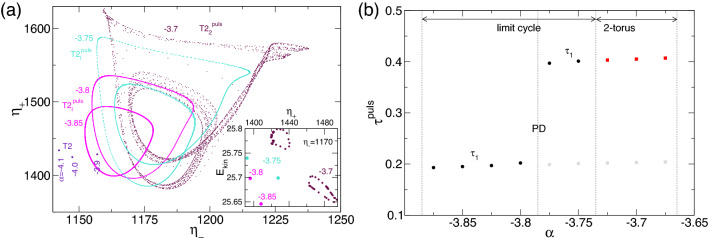
Phase space and period times for T2$^\text {puls}$. (**a**) Phase portraits of T2$_1^\text {puls}$ and T2$_2^\text {puls}$ states for $\alpha$, as indicated on $(\eta _-,\eta _+)$. The inset shows the corresponding two-dimensional Poincaré sections, $(E_{kin},\eta _+)$, with $\eta _-=1,170$. (**b**) Variation with $\alpha$ in time period $\tau ^\text {puls}$ for T2$^\text {puls}$. Numbers in the figure identify the strength of radial flow, $\alpha$. Gray-filled symbols highlight the underlying basic period.

Figure 8 shows a phase portrait of $(\eta _-,\eta +)$, illustrating the evolution of T2 solutions from T2$_1^\text {puls}$ to T2$_2^\text {puls}$. The inset gives the corresponding two-dimensional Poincaré section for $\eta _-$ on $(E_{kin},\eta _+)$. The Poincaré section corresponds to $\eta _-=1,170$. In the phase portrait, $(\eta _-,\eta +)$, points at $\alpha \in \{-4,1,-4,-3.9\}$ show the stationary T2 state, whereas the curves for $\alpha \in \{-3.85,-3.8,-3.75\}$ correspond to the limit cycle solution, T2$_1^\text {puls}$, and finally the 2-torus of T2$_2^\text {puls}$ at $\alpha =-3.7$. In the corresponding Poincaré section, limit cycle solutions appear as single points [i.e., two points for the T2$_1^\text {puls}$ solution after the PD at $\alpha \approx -3.77$ and the 2-torus as two (almost) closed curves]. After the PD, the phase portrait shows a doubled curve corresponding to the appearance of half the former frequency, $\omega _1/2$ (Fig. 6). The crucial differences in $\eta _+$ and $\eta _-$ regarding the distance to line $\eta _=\eta _+$ is a measure of the degree to which $Z_2$ symmetry is broken (see cf. (M)RWs in Fig. 4).

#### Propagating flow structures

**Figure 9:**
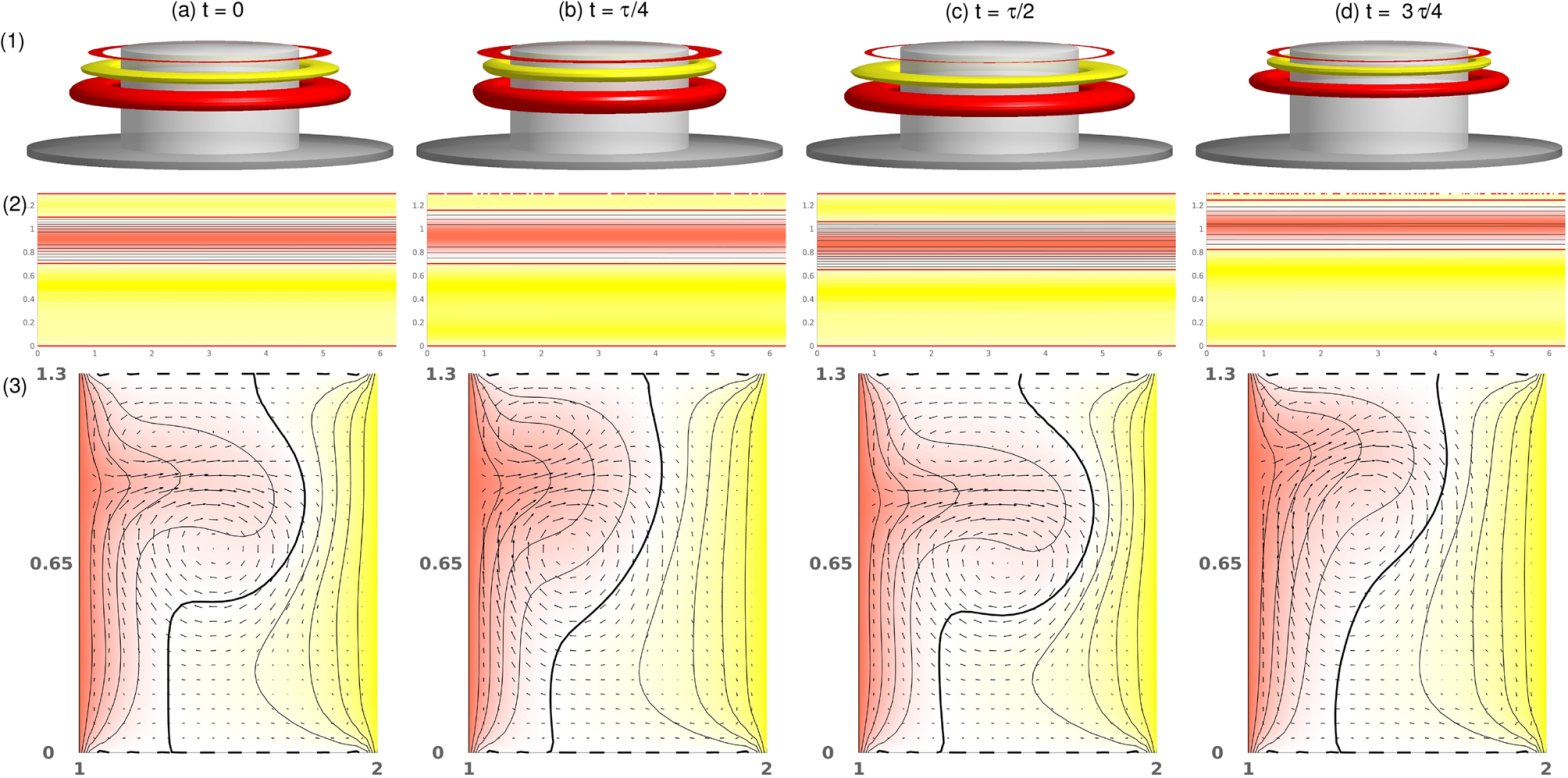
Visualization of propagating flow state pV$_1$ for $\alpha =0$, as shown in Fig. 5 with isosurfaces $\eta =\pm 240$. The propagating period is $\tau \approx 0.641$. Also see the movie, movie_pV1_alpha0.avi, in SMs.

The pV states discussed here have higher complexity than the ones in previous works^27,28^. Currently, all observed pVs in classical fluids are relatively simple limit cycle solutions (1-torus). However, this does not hold for the pVs presented here. Moreover, most of ours do not illustrate a pure propagation of vortices with generation and annihilation. Most are combinations of propagating flow structures and additional pulsations. However, the pulsations are similar to the previously described ones for T2$^\text {puls}$ solutions. Notably, more complex pV dynamics have been observed for ferrofluids under the influence of external magnetic fields^29,30^.

**Figure 10:**
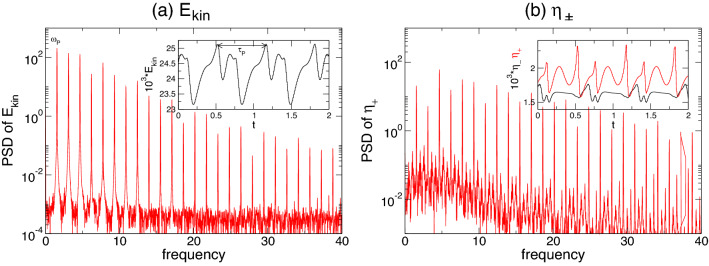
Time series and power spectral densities (PSD) of the pulsating flow state, pV$_1$ for $\alpha =0$. PSDs of (**a**) $E_{kin}$ and (**b**) $\eta _+$ for the pV$_1$ state. The propagation/period time (period of vortex generation and annihilation) is $\tau _P\approx 0.641$ with corresponding frequency $\omega _P\approx 1.56$. Insets show time series of $E_{kin}$, $\eta _+$ [red], and $\eta _-$ [black].

Stable pV states appear for both negative and positive radial flows in the parameter range, $-1.411\leqslant \alpha \leqslant 2.05$ (Fig. 2). However, some crucial differences from earlier observations exist^27,28^. First, although they are axisymmetric, our pVs are *not* $Z_2$ symmetric. Second, our pV states appear stable in two *separated* regions, $-1.411\leqslant \alpha \leqslant 0.15$ (region A) and $0.67\leqslant \alpha \leqslant 2.05$ (region B) (Fig. 2), leaving a region in-between in which pVs cannot be found. Notably, all efforts to stabilize them failed. All restrictions to axisymmetry ($m=0$ mode) subspaces (see section “Unstable pV states”) resulted in the N2 stationary flow state. Third, behaviors related to variations in $\alpha$ crucially differ within regions A and B. On one hand, in region A with (mainly) negative $\alpha$ pVs, a relaxed character typical of slow–fast dynamics appears, which eventually ends in a saddle node on an invariant circle (SNIC) bifurcation^37,38^. On the other hand, in region B with positive $\alpha$, more complex bifurcation sequences appear. However, the underlying dynamics seem to be associated with the same slow–fast dynamics (Fig. 14). This can be observed visually, but the given parameter range is too small to offer more quantitative analyses.

**Figure 11:**
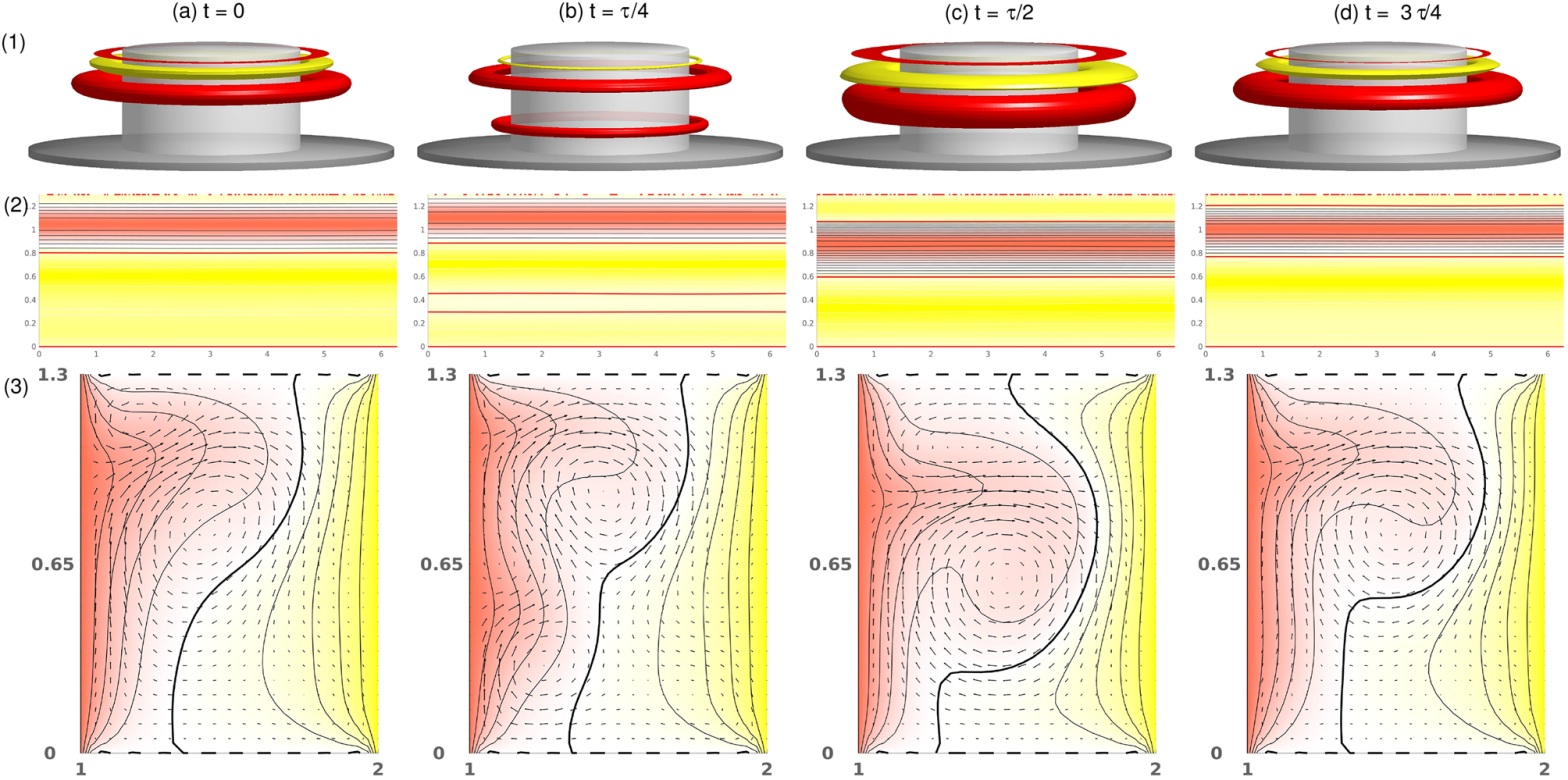
Visualization of the propagating flow state, pV$_2$, for $\alpha =2$. As shown in Fig. 9 with isosurfaces of $\eta =\pm 240$, the propagation period is $\tau _P\approx 0.405$. See also movie, movie_pV2_alpha2.avi, in SMs. Note the clearly visible appearance of the second propagating vortex pair at (*b*) $t=\tau /4$.

Although the flow dynamics appearing in the pV states within the two separated regions, A and B, are largely identical, there are some important differences. A and B regions (Fig. 2) both emanate secondary jets from bottom to top, which merge with their former respective outward directed jets. The inner boundary layer is dominant, whereas the outer boundary layer mainly responds to the dynamics of the inner one. In region A, we find only pV$_1$ (Figs. 9 and 10), which is a limit cycle solution. Instead, in region B, pV$_2$ (Fig. 11) and pV$_3$ are found, representing 2- and 3-torus solutions. We see that for radial outflow $\alpha >0$ (region B), as expected, the entire flow dynamics is moved outward (see also additional Figs. 3 and 4 in SM) into a wider region of the bulk interior. As a result, the outer boundary layer becomes smaller while remaining passive and responding to the inner one.

**Figure 12:**
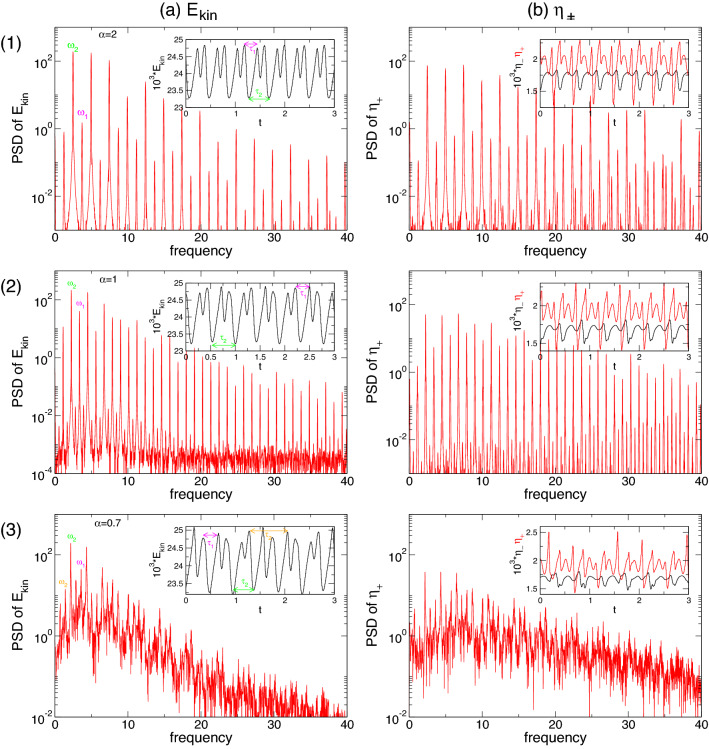
Time series and power spectral densities (PSDs) of pVs for different $\alpha$. PSDs of (**a**) $E_{kin}$ and (**b**) $\eta _+$ for pVs: (1) $\alpha =2$ with period time $\tau _P\approx 0.405$ and corresponding frequency $\omega _P\approx 2.47$; (2) $\alpha =1$ with period time $\tau _P\approx 0.896$ and corresponding frequency $\omega _P\approx 1.116$; (3) $\alpha =0.7$ with period time $\tau _P\approx 1.395$ and corresponding frequency $\omega _P\approx 0.717$. Insets show time series of $E_{kin}$, $\eta _+$ [red], and $\eta _-$ [black], respectively.

The PSDs in Fig. 6 highlight the increasing complexity of the pV in region B with decreasing $\alpha$. The PSDs for pV$_2$ at $\alpha =2$ and $\alpha =1$ (Fig. 6(1,2)) indicate the 2-torus characteristics with clearly visible frequencies, $\omega _i, \,i\in \{1,2,3\}$, which also highlight the PD. $\omega _1$ is associated with period $\tau _1$ of one vortex propagation (bottom to top), whereas $\omega _2$ corresponds to $\tau _2$, incorporating the period time for one single vortex propagation plus pulsation. With the appearance of the third incommensurate frequency, the corresponding PSD of pV$_3$ at $\alpha =0.7$ (Fig. 6(3)) indicates only the main underlying frequency, $\omega _1$, associated with the vortex propagation, and $\omega _2$ associated with the overall combination of propagation and pulsation.

**Figure 13:**
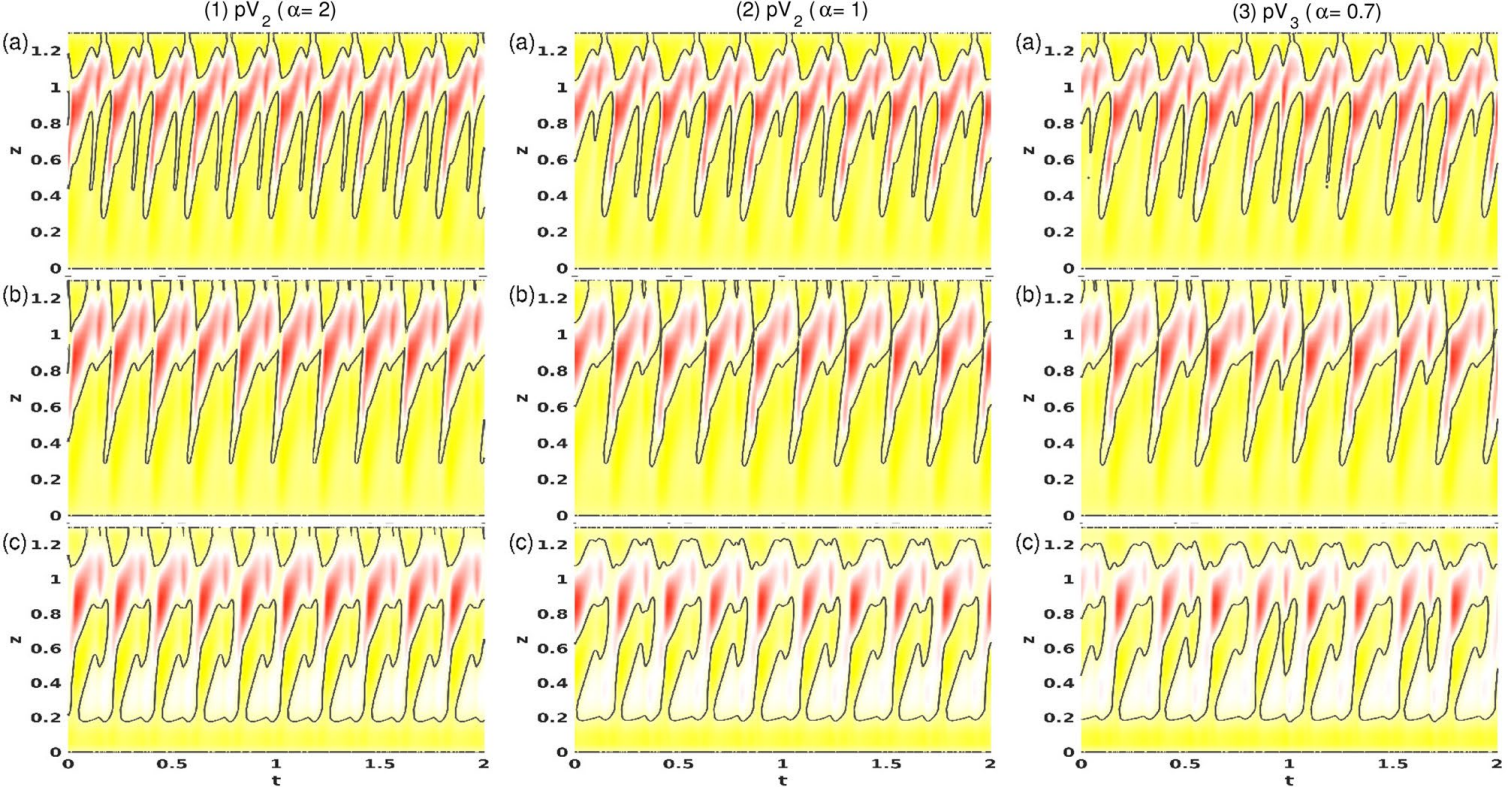
Spacetime plot of *u* for pV$_2$ at (**1**) $\alpha =2$, (**2**) at $\alpha =1$, and for pV$_3$ at (**3**) $\alpha =0.7$. Visualizations are shown for three different radial positions, (a) $r=d/4$, (b) $r=d/2$, and (b) $r=3d/4$. Red (dark gray) and yellow (light gray) correspond to positive and negative values, respectively (see Fig. 7).

The spacetime plots in Fig. 13 illustrate the flow dynamics dominated by the two main characteristics: propagation and pulsation. First, the propagation given by the periodic new generation, (upward) propagation and elimination of one vortex pair, can be seen by the periodic re-appearing inclined (black) zero contour lines (Fig. 13b½). Second, the pulsation is visible by the wave/belly-like modulation of the zero-contour line at $y\approx 0.8$ in Fig. 13 between two of the previously described propagation cycles. Comparing Figs. 13(1) ($\alpha =1$) and (2) ($\alpha =2$), the doubled period after the PD is clearly visible. With the appearance of a third incommensurate frequency and the appearance of the 3-torus solution, the spacetime plot shows increased complexity without any obvious period [Fig. 13(2) ($\alpha =0.7$)]. The loss of periodicity can be best seen at midgap $(b)=d/2$ close to the upper lid. In the case of (2) pV$_2(\alpha =1)$, one sees the periodic (repetitive) appearance of a small *island* in each second tong when the propagating vortex collides with the upper lid, but this periodicity is lost for (3) pV$_3(\alpha =0.7)$. For pV$_3(\alpha =0.7)$, these islands can be appeared irregularly, both in size and time (not every period).

Note, one might expect that the solution(s) pV$_{l,2}$ appears out of a stationary solution as does T2$_{l,2}^{puls}$ do out of T2. In particular, this should be an analogous asymmetry solution with two vortices. However, we could not find such an asymmetric stationary flow, neither stable or unstable.

**Figure 14:**
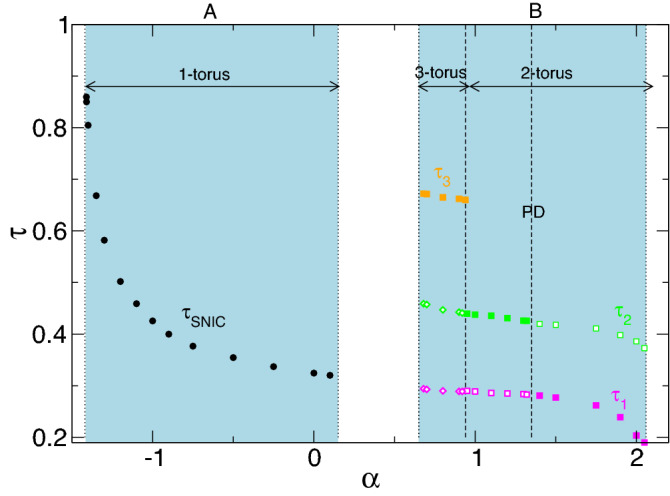
Variation of $\tau$ with radial flow $\alpha$ for stable pV states. Note that in region $0.15<\alpha <0.67$, pVs do not appear. Filled symbols highlight the basic period (see PSDs in Fig. 12).

The different appearance times, which can be identified within the different flow states, pV$_1$, pV$_2$ and pV$_3$ (in regions A and B), are illustrated in Fig. 14. Always detectable is the underlying minimal period time, which, in region A, is indicated as $\tau _\text {SNIC}$. It significantly increases with decreasing $\alpha$, as discussed next (see cf. PSDs in Fig. 12).

### Vortex break down

**Figure 15:**
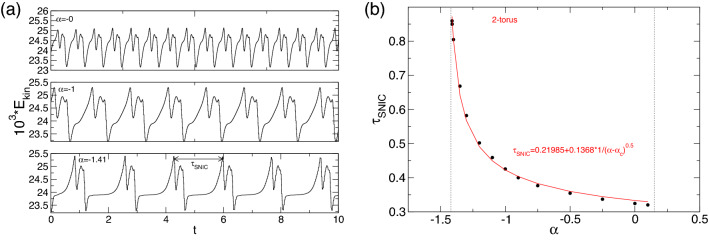
Saddle node on an invariant circle bifurcation for pV$_1$. (**a**) Time series of $E_{kin}$ for pV$_1$ at $\alpha$ as indicated; (**b**) Variation of $\tau _\text {SNIC}$ with $\alpha$ for pV$_1$ (see Fig. 14). The solid circles are the computed periods, and the line is a fit of the form, $\tau _\text {SNIC}=a_0+a_i/\sqrt{\alpha -\alpha _c}$. Note that in the region, $0.15<\alpha <0.67$, *no* pV state, whether stable nor unstable, can be detected, even in subspaces (see section “Unstable pV states”).

The characteristics of pVs are quite different in both separated regions A and B. In region A (pV$_1$,), it changes rapidly with variations in $\alpha$. For $\alpha \in (-1.411,0)$, Fig. 2 shows that $E_{kin}$ decreases with more negative $\alpha$ (inflow) towards an asymptotic value. Here, the flow shows a relaxation character type typical of slow–fast dynamics. The long period associated with $1/\omega _\text {SNIC}$ (i.e., $\tau _\text {SNIC}$), is clearly evident (Fig. 15). At about $\alpha =-1.412$, the limit cycle ceases to exist as the frequency associated with the vortex regeneration (generation and annihilation), $\omega _\text {SNIC}$, goes to zero, and the associated period, $\tau _\text {SNIC}$, becomes unbounded.

Figure 15a shows the time series of $E_{kin}$ for pV$_1$ at three different $\alpha \in \{-1.411,-1,0\}$, indicating the increase in period time for pV$_1$. Corresponding variations with $\alpha$ of $\tau _\text {SNIC}$ for pV$_1$ are shown in Fig. 15b. As $\alpha \rightarrow \alpha _c \approx -1.415$ from above, $\tau _\text {SNIC} \rightarrow \infty$, following the $1/\sqrt{\alpha -\alpha _c}$ scaling associated with the SNIC bifurcation^37,38^. Circles represent the computed periods, and the line fits the form, $\tau _\text {SNIC}=a_0+a_i/\sqrt{\alpha -\alpha _c}$. At the bifurcation, a saddle and node are created, which are connected via two heteroclinic curves forming an invariant circle that ceases to exist after bifurcation. In the present scenario, the system falls back to a stationary, stable solution, N2, which is found after bifurcation, which stably coexists with MRW$_{1,2}$; see Fig. 2.

In region B, stable pV states between $0.67\leqslant \alpha \leqslant 2.05$ show more complex dynamics. Here, the simplest pV state already includes *two incommensurate* frequencies describing a 2-torus solution (pV$_2$). Starting at the largest value, $\alpha \approx 2.02$ in pV$_2$, and decreasing $\alpha$, we detect the following sequence. First a PD bifurcation occurs at $\alpha \approx 1.46$. The newly doubled period is clearly visible in the corresponding time series, and the PSD of $E_{kin}$ is presented in Fig. 12b. With the continuously decreasing $\alpha$, a third incommensurate frequency, $\omega _3$, appears at $\alpha \approx 0.98$, generating the 3-torus solution, pV$_3$. Interestingly, the period of this flow is about three times ($\omega \approx 1/3$) the one of the underlying basic state (see Fig. 14). The 3-torus eventually loses stability at $\alpha \approx 0.67$ when the flow transient returns to the stationary N2 state.

Although the global dynamics is significantly more complex, it is important to understand that similar relaxation types typical of slow–fast dynamics can also be observed/anticipated in region B. With decreasing $\alpha$, the corresponding (double) period times for pV$_2$ and pV$_3$ increase similar to the behavior described above ($\tau _\text {SNIC}=a_0+a_i/\sqrt{\alpha -\alpha _c}$). However, this is mainly a qualitative observation, as the region is quite narrow, making quantitative analysis too complicated. Note that the time series of $E_{kin}$ ($\eta _\pm$) and corresponding PSDs for pV$_2$ and pV$_3$ in region B (Fig. 12) are significantly different and more complex than those of pV$_1$ in region A (Figs. 10 and 15a), which lead to the SNIC bifurcation.

**Figure 16:**
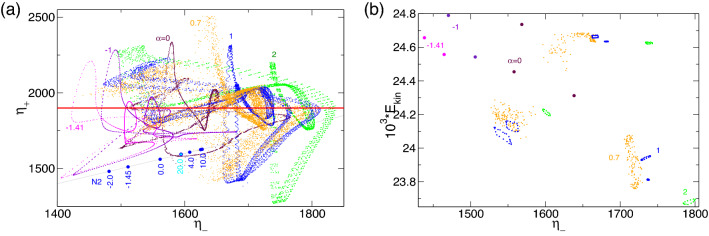
(**a**) Phase portraits of pV$_1$, pV$_2$ and pV$_3$ states for $\alpha$ as indicated on $(\eta _-,\eta _+)$ and (**b**) the corresponding two-dimensional Poincaré sections, $(E_{kin},\eta _-)$, with $\eta _+=1900$ (horizontal red line in (**a**)). Numbers in the figure identify the radial flow, $\alpha$. The thin gray line in (*a*) indicates the diagonal $\eta _-=\eta _+$, upon which all $Z_2$ symmetric solutions (i.e., N2) can be found.

Spacetime plots (Fig. 13), phase portraits, and Poincaré sections (Fig. 16) clearly highlight the complexity of the pV solutions. However, crucial bifurcation sequences (e.g., PD on pV$_2$) at $\alpha \approx 1.34$ can be seen in Fig. 14. Regarding (M)RWs (Fig. 4) and T2$^\text {puls}$, the broken $Z_2$ symmetry is visible by the distance of the phase portraits to the (gray) line, $\eta _=\eta _+$.

### Unstable pV states

To shed light on pVs and their origins, we continued this solution branch for larger $\alpha$ after it became unstable at $\alpha \approx 2.07$. One would expect the pV states to appear out of a stationary solution, like the T2$^\text {puls}$ state, which arises out of the T2 state. For pVs, one could speculate that this is an analogous asymmetric stationary solution with two vortices, like the symmetric pV appearing out of the nV^27^. The single-cell anomalous mode, A1, is a candidate typically found in direct competition with N2^26,31^. As discussed, pV states are axisymmetric (i.e. pure $m=0$ modes); thus, we continued our investigation restricted to the $m=0$ symmetry subspace so that we could follow the unstable pVs and detect more complex flow dynamics.

**Figure 17:**
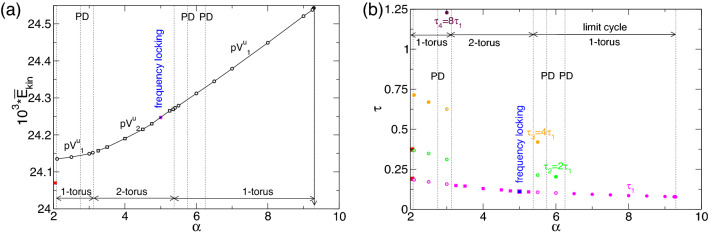
Time dependency of unstable pV$^u$ with $\alpha$.Variation of (**a**) $E_{kin}$ and (**b**) $\tau$ with $\alpha$ for unstable pV$^u$ solutions. PD indicates period-doubling bifurcations. The vertical arrow in (**a**) illustrates a transition towards the stationary N2 state [outside the presented parameter range (smaller $E_{kin}$; see also Fig. 2). Colored symbols in (**b**) refer to the different period times $\tau _i, i\in \{1,2,3,4\}$, respectively, which appear throughout the periodic doubling bifurcations (PD) as multiples of the basic period time $\tau _i$. Thus, these are all 1-torus, that are limit cycle solutions. The filled symbols show the actual period time $\tau _p$ of the corresponding solution, while open symbols illustrate the still underlying time(s) at the bifurcating parameter.

Figure 17a presents the variation with $\alpha$ for the kinetic energy, $E_{kin}$, and period time, $\tau$, for unstable pV$^u$ solutions. Although, for stable pV states (Fig. 2), $E_{kin}$ decreases with increasing $\alpha$, for unstable pV$^u$ states, $E_{kin}$ behaves differently, increasing with the increasing $\alpha$. The final detected stable pV$_2$ state at $\alpha \approx 2.07$ is a 2-torus solution [Fig. 12(3) and 14]. For slightly larger $\alpha$ [leftmost detected solution in Fig. 17a], the flow dynamics appear as a limit cycle solution, pV$^u_1$. We believe that with decreasing $\alpha$, a second saddle collides at $\alpha \approx 2.075$, introducing a second incommensurate frequency and transferring stability from the previous unstable pV$^u_1$ to the next stable (in subspace $m=0$) pV$^u_2$. In the other direction, starting with pV$^u_1$ at $\alpha =2.1$ and with an increasing $\alpha$, the flow first undergoes a PD bifurcation at $\alpha \approx 2.74$ prior to the appearance of the incommensurate frequency at $\alpha \approx 3.125$, which renders the flow (pV$^u_2$) to exist on a 2-torus invariant manifold. At an $\alpha = ~5$, we detect frequency locking on the 2-torus solution, pV$^u_2$, prior to the incommensurate frequency disappearing at $\alpha \approx 5.37$, again leaving a limit cycle solution, pV$^u_1$, behind. As $\alpha$ continues to increase, pV$^u_1$ twice undergoes a backward PD bifurcation at $\alpha \approx 5.76$ and $\alpha \approx 6.24$ before it eventually disappears at $\alpha \approx 9.95$ and moves transiently to the stationary state, N2 (indicated by vertical arrow in Fig. 17a). For larger $\alpha$, all efforts failed to follow pV$^u_1$, and we cannot find an asymmetric stationary flow (e.g. A1, single-cell anomalous mode), neither stable nor unstable, as initially expected. It is worth mention that the non-detection of a connection to a basic state does Not mean that such a connection does not exist. Through investigating of various parameters in the Taylor–Couette system, e.g. $Re_i$, $Re_o$, $\Gamma$, $\eta$, ..., it may allow many different possibilities or connections.

**Figure 18:**
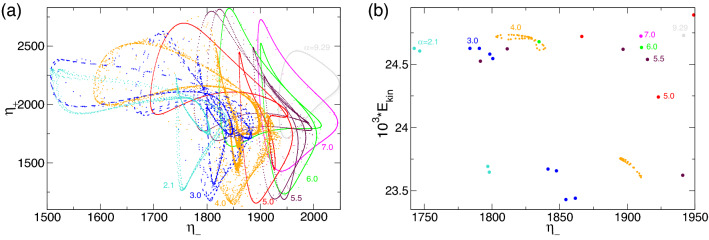
(**a**) Phase portraits of unstable pV states for $\alpha$ as indicated on $(\eta _-,\eta _+)$ and (**b**), the corresponding two-dimensional Poincaré sections, $(E_{kin},\eta _-)$ with $\eta _+=1900$. Numbers in the figure identify the strength of radial flow, $\alpha$.

**Figure 19:**
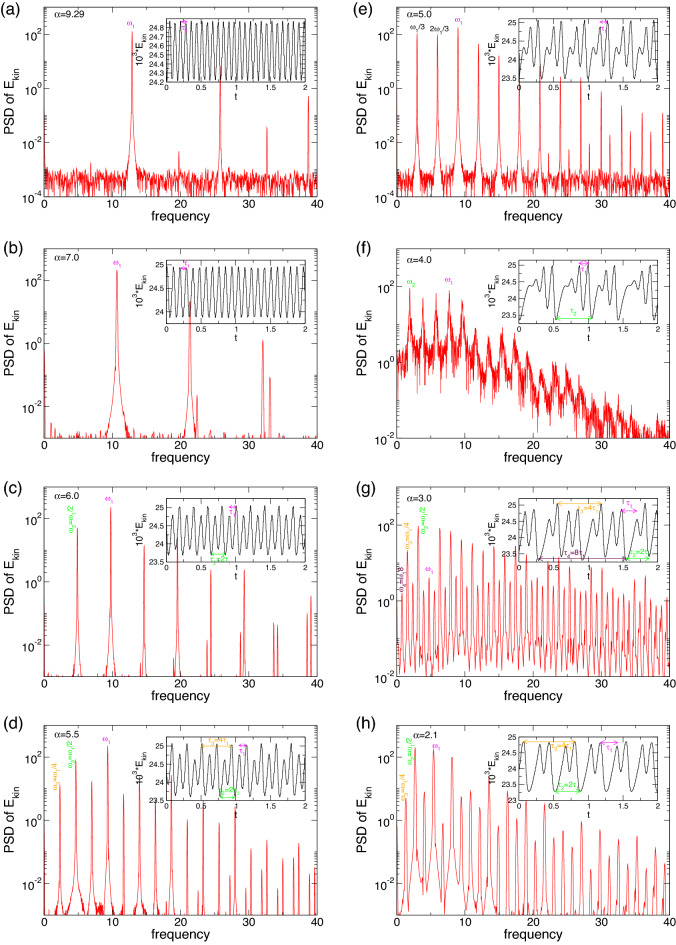
Time series and power spectral densities (PSDs) of $E_{kin}$ of unstably propagating flow states, pV$^u$, for different $\alpha$, as indicated. Insets show time series of $E_{kin}$. In both, dominant times and frequencies, respectively, are highlighted. Note that all PSDy belong to limit cycle solutions (see Fig. 17) but (*f*) presents a 2-torus solution.

The observed pinning phenomenon at $\alpha \approx 5$ bears some resemblance to the frequency-locking phenomena. In the frequency-locking case^39–42^, the system contains two non-zero frequencies, whereas their ratio becomes constant in a certain region of the parameter space (i.e., the resonance horn). As a result, the system appears to be characterized by a single frequency, as in the case presented here. By appearing on the 2-torus, the moment of pinning the phase-space solution shows a single curve characteristic for a 1-torus limit cycle solution (see Figs. 18 and 19e).

Figure 17b illustrates the different underlying times, $\tau _i$, and frequencies, $\omega _i$, for pV$^u_i$ ($i\in \{1,2,3\}$) as they are detected in the different solutions with variations in $\alpha$. Colored symbols in Fig. 17 refer to the different period times $\tau _{1,2,3,4}$, respectively, which appear throughout the periodic doubling bifurcations (PD). The filled symbols show the actual period time $\tau _p$ of the corresponding solution, while open symbols illustrate the still underlying half-period time at the bifurcating parameter. As shown for stable pV states, the basic time $\tau _1$ (frequency, $\omega _1$), corresponding to one period of vortex generation and annihilation, is present throughout all solutions, although with increasing complexity in PSDs (Fig. 19).

Figure 18 shows the phase portrait on $(\eta _-,\eta _+)$ and a corresponding two-dimensional Poincaré section, $(E_{kin},\eta _-)$, with $\eta _+=1900$ of selected unstable pV$^u$s (limit cycle pV$^u_1$ and 2-torus solution pV$^u_2$) at $\alpha$, as indicated. Compared with the phase portrait of stable pVs in Fig. 16a, it is less complicated, as most except pV$^u_2$ at $\alpha =4$ represent limit cycle solutions. The 2-torus state, pV$^u_2$, at $\alpha =4$ is evidenced by the closed loop structure of the Poincaré section (Fig. 18b). The phase portrait (Fig. 18a) indicates that no solution is symmetric.

Figure 19 shows the corresponding PSDs, which illustrate the different frequencies involved in the solution. The PSDs for $\alpha =7,6,$ and 5.5 (Fig. 19b–d) show a doubling period beginning from the basic one, $\tau _1$. For $3.125\lesssim \alpha \lesssim 5.375$, the flow describes a 2-torus solution with two incommensurate frequencies, resulting in more complex PSD (Fig. 19e). However, at $\alpha =5$, all frequencies are locked to multiple s of the basic one, $n\times \omega _1, \,n\in \{N\}$.

Note that we tried the same approach by restricting simulations to an $m=0$ symmetry subspace to follow the pV$^u$ states in the parameter range between the stably existing pVs in regions A and B. However, despite all efforts, we could not detect any stable or unstable pV solutions for these parameters. All simulations ended in the basic stationary N2 solution. This led us speculate that either pVs are more complex in the sense that higher azimuthal modes are involved, and therefore, their axisymmetry is broken, or the basin of attraction for pVs becomes very small and/or collides with those of other solutions, resulting in N2. However, this question can’t cannot be answered here and will be the subject of future investigations.

Moreover, for $\Gamma \geqslant 4$, it has been shown that the $Z_2$ symmetric and axisymmetric propagating pV bifurcates out of a stationary solution with a corresponding number nV of vortices^27^ in variations of $Re_i$. For $\Gamma =4$, a pV with six propagating vortices appeared out of a stationary solution with 6N. However, for short aspect ratio systems, $\Gamma =1.3$, we may get different scenario. Despite all efforts, we could not be able to detect stable or unstable connections between pVs and other stationary solutions. Instead, pVs appeared as isolated and stable in different disconnected regions. For main reason of not detecting a connection to a stationary solution, it might be originated in the broken $Z_2$ symmetry, which is contrast to the pV findings of earlier studies^27^.

## Discussion and conclusion

As a fundamental paradigm of fluid dynamics, TCSs have been extensively studied for more than a century, first experimentally and later computationally. The present work, considering numerical simulations for small-aspect-ratio wide-gap counter-rotating TCSs, revealed various complex flow solutions as the radial flow changed. For given parameters, the dynamics in absence of any radial flow were dominated by competition between the two-cell normal mode (N2), RWs, and the single-cell anomalous mode (A1). Although the N2 (axisymmetric, pure $m=0$ mode) exists stably over the investigated parameter range, $-5\leqslant \alpha \leqslant 100$, the RWs, together with their more complex cousins, MRWs, appear stable over a wide range of $\alpha$, and both RW and MRW are non-axisymmetric in the $m\ne 0$ mode.

In addition to previously known pVs, we observed new *axisymmetric* and *time-dependent* solutions with variations in $\alpha$. These were either pure pulsating flow structures (T2$^\text {puls}$) or propagating vortices (pVs), the latter with and without *pulsation*. The pulsating flow structure, T2$^\text {puls}$, appears via a supercritical Hopf bifurcation out of a stationary twin-pair flow, T2. As N2 and T2 states are axisymmetric (pure $m=0$ mode), and T2 is similar to earlier observed twin-pair states^31^, it has a broken $Z_2$ symmetry. With increasing $\alpha$, the stationary T2 solution underwent a sequence of Hopf bifurcations alongside PD bifurcations, with the appearance of a second incommensurate frequency that modified the solution from a limit cycle, T2$_1^\text {puls}$, to a 2-torus T2$_2^\text {puls}$. All solutions presented symmetry degenerated by reflection about the system mid-height $(\text {T2}^\text {puls}_{1,2})^*=K_z\text {T2}^\text {puls}_{1,2}$. The same held for the stationary state, T2$^*=K_z$T2. Usually with TCS, the appearance of a secondary frequency is connected to the stimulation of higher, non-axisymmetric modes, $m\ne 0$^26^. This does not apply for $T2^\text {puls}_2$. Instead, the secondary incommensurate frequency appears as a modulation atop the primary one ($\approx \omega /4$). In the case of T2$_2^\text {puls}$, a variation of the strength/intensity of the pulsation results.

Regarding the propagating vortices, we observed new, axisymmetric pVs that differed from earlier pVs discussed in the literature^27,28^ as they have broken $Z_2$ symmetry. Here, the detected pVs were (quasi-) periodic solutions generated by the shear flow near one of the Ekman cells induced by the non-rotating end walls. Thereafter, these axisymmetric vortices propagated through the bulk from its place of birth near one Ekman cell towards the opposite end of the system, where they eventually annihilated in the Ekman cell. As a result, pVs existed as degenerated solution about the reflection at system mid-height, pV$^*=K_z$pV. Additionally, with variations in $\alpha$, pVs can change from pure propagating flow structures and evolve into a combination of propagating and oscillating structures. We found pVs to exist stably in two different *separated* parameter regions, A ($-1.411\leqslant \alpha \leqslant 0.15$) and B ($0.67\leqslant \alpha \leqslant 2.05$). In both regions, the flow dynamics were dominated by the propagating vortices with their generation and annihilation; however, the evolution of the variation in $\alpha$ was crucially different. In region A, the pV$_1$ state appeared only as a 1-torus (limit-cycle) solution, showing typical characteristics of slow–fast dynamics, eventually evolving into a SNIC bifurcation. These, in small-aspect-ratio TCS flows, have been observed experimentally and numerically between a symmetric 2-torus (modulated rotating waves) and a symmetrically related 1-torus (rotating waves)^25,43,44^. Typical SNIC bifurcations on a symmetric 2-torus have saddles and nodes as conjugate pairs of a 1-torus (rotating waves), whereas more recently, a higher dimensional SNIC bifurcation was described, taking place on the 3-torus, leaving two 2-torus states behind^38^. In region B, the dynamics were more complex as an intrinsic second incommensurate frequency was present, causing the flow state, pV$_2$, to live on a 2-torus invariant manifold. Moreover, with variations in the radial flow, $\alpha$, we detected a period-doubling bifurcation and a third incommensurate frequency, $\approx \omega /3$, which resulted in a 3-torus solution, pV$_3$.

Using axisymmetry ($m=0$ mode) restricted the subspace in our simulations, unveiling additional complex underlying flow dynamics, including a versatile PD and Hopf bifurcation scenarios and frequency-locking limit cycles and 2-torus solutions. The pulsating flow states, T2$^\text {puls}$, propagating vortices, and pVs (stable and unstable) presented symmetry degenerated via reflections about the system mid-height: $(\text {pV}^{(u)}_i)^*=K_z\text {pV}^{(u)}_i$, $i\in \{1,2,3\}$. That is, by presence of incommensurate frequencies, it can be appeared as 1-, 2- and 3-torus solutions, which is known as the Ruelle–Takens–Newhouse route to chaos. Therefore, the observed bifurcation scenario is the Ruelle-Takens-Newhouse route to chaos and the period doubling bifurcation, which exhibit rich and complex dynamics.

Similar bifurcations, including low-dimensional tori and even strange attractors, have been reported elsewhere with chaotic or turbulent flows. It is believed that these bifurcations, either *symmetry breaking* or *symmetry restoring*, may play important roles in the transition to turbulence and the general characterization of turbulent states^45^. It has also been suggested that reversals of the geomagnetic poles may be explained in terms of symmetry-breaking SNIC bifurcations in a turbulent setting^46^.

TCS flows at small aspect ratios are a good paradigm for studying symmetry-breaking bifurcations and more complex low-dimensional objects, including invariant tori and attractors. In particular, the study of additional radial flows in narrow aspect-ratio systems with increased system complexity are important for astrophysical studies. Although very specialized, the results presented here may have implications for drag reductions by suction, accretion in astrophysical disks, and perhaps in the flow in of the earth’s polar vortex.

## Supplementary Information

Supplementary Information.

Supplementary Movie 1.

Supplementary Movie 2.

Supplementary Movie 3.

Supplementary Movie 4.

Supplementary Movie 5.

## Data Availability

The datasets that support the results of this research work are available from the author (S.A.) on reasonable request.
