# Supplementary material for: Bifurcation phenomena in Taylor–Couette flow in a very short annulus with radial through-flow
Source: Sci Rep. 2022 Dec 21;12:22113. doi: 10.1038/s41598-022-26645-6 (PMC9772330; doi:10.1038/s41598-022-26645-6)
Supplement: Supplementary file 1 — Supplementary Information. [file 41598_2022_26645_MOESM1_ESM.pdf]

Supplementary Materials to
"Bifurcation phenomena in Taylor-Couette flow in a very short annulus with radial through-flow"

Sebastian Altmeyer
Castelldefels School of Telecom and Aerospace Engineering,
Universitat Politècnica de Catalunya, 08034 Barcelona, Spain

M. Sankar
Department of General Requirement, University of Technology and Applied Sciences, Ibri-516, Sultanate of Oman

Younghae Do
Department of Mathematics, Nonlinear Dynamics & Mathematical Application Center,
Kyungpook National University, Daegu 41566, Republic of Korea

I. NUMERICAL METHOD

The Navier–Stokes equations,

$$\partial_t \mathbf{u} + (\mathbf{u}\cdot\nabla)\mathbf{u} = -\nabla p + \nabla^2 \mathbf{u}, \qquad \nabla\cdot\mathbf{u} = 0, \tag{1.1}$$

are solved using a second-order time-splitting method with consistent boundary conditions for pressure [1, 2]. Our code, G1D3 [3], is a combination of a finite difference method in the radial and axial directions (r, z) and a Fourier–Galerkin expansion in the azimuthal direction (θ) with explicit time splitting, resulting in a decomposition,

$$f(r,\theta,z,t) = \sum_m f_m(r,z,t)\, e^{im\theta} \tag{1.2}$$

of all fields, $f \in \{u, v, w, p\}$. For the parameter regime considered here, the choice, $m_{max} = 10$, provides adequate accuracy. We use a uniform grid with spacing $\delta r = \delta z = 0.02$ and time steps $\delta t < 1/3,800$. For diagnostic purposes, we also evaluate the complex mode amplitudes, $f_{m,n}(r,t)$, obtained from a Fourier decomposition in the axial direction.

$$f_m(r,z,t) = \sum_n f_{m,n}(r,t)\, e^{inkz}. \tag{1.3}$$

At the junctions where the stationary endwalls meet the rotating cylinders, the idealized boundary conditions are discontinuous, wheras in experiments there are small but finite gaps at these junctions where the azimuthal velocity adjusts to zero. To overcome this problem, a regularization of the discontinuous idealized boundary conditions is implemented of the form

$$v(r,\theta,\pm 0.5\Gamma,t) = Re_i \exp([r_i - r]/\epsilon) + Re_o \exp([r - r_o]/\epsilon), \tag{1.4}$$

where ϵ is a small parameter that characterizes the physical gaps [4]. For all solutions reported here, we have used $\epsilon = 6\times 10^{-3}$.

Further, as a global measure to characterize the different flow structures, we use the total modal kinetic energy,

$$E_{kin} = \sum_m E_m = \frac{1}{2}\sum_m \int_0^{2\pi}\int_{-\Gamma/2}^{\Gamma/2}\int_{r_i}^{r_o} \mathbf{u}_m \mathbf{u}_m^* r\,\mathrm{d}r\,\mathrm{d}z\,\mathrm{d}\theta, \tag{1.5}$$

where $\mathbf{u}_m$ ($\mathbf{u}_m^*$) is the mth (complex conjugate) Fourier mode of the velocity field. Thus, for axisymmetric solutions, such as stable cylindrical Couette flow or Taylor vortex flows, only E_0 is non-zero.

II. FLOW STRUCTURES AND DYNAMICS

A. Mode amplitudes

Figure S1 shows mode amplitudes, $|u_{m,n}|$ (Eq. (1.3)), of different flow states as indicated (see Fig. 3 in main manuscript. The mode spectra highlight the different solution characteristics. The $m = 2$ mode in (c) clearly identifies RW_2 (here mode $m = 1$ is equal to zero), whereas both $m = 1$ and $m = 2$ modes in (d) identify $\mathrm{MRW}_{1,2}$. The spectra, $m - n$, of N2 and T2 only contain $m = 0$, illustrating the axisymmetry of these solutions. However, it does not give any further information about other symmetries (see the main manuscript).

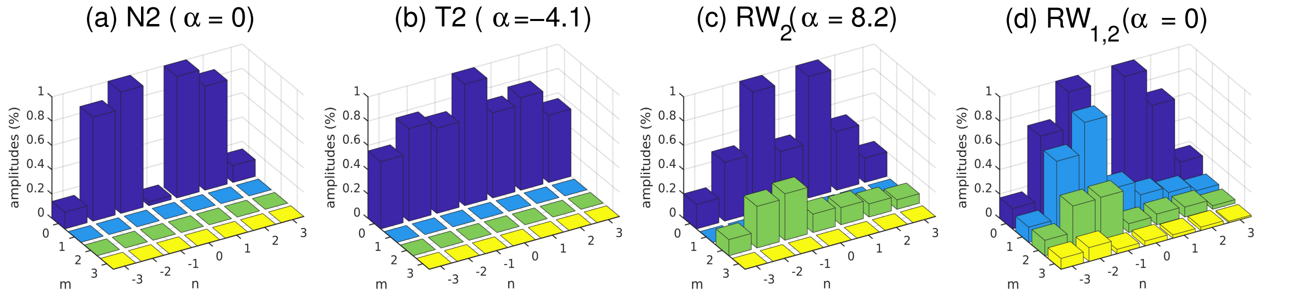

FIG. S1: **Mode amplitudes for stationary and rotating flow structures.** Mode amplitudes $|u_{m,n}|$ (Eq. (1.3)) of the radial velocity field, u, above the $m-n$ plane. Different stationary and rotating flow structures with radial flow α (see Fig. 3 in the main manuscript), as indicated above. The values are scaled so that the maximum mode amplitude equals one (N2: $|u_{0,1}| = 1$).

B. Flow visualizations

Figures S2, and S3 illustrate the propagating vortex structures of pV$_2$, as they appear for different radial outflow $\alpha > 0$ region B. (see also main manuscript Fig. 9 and 11).

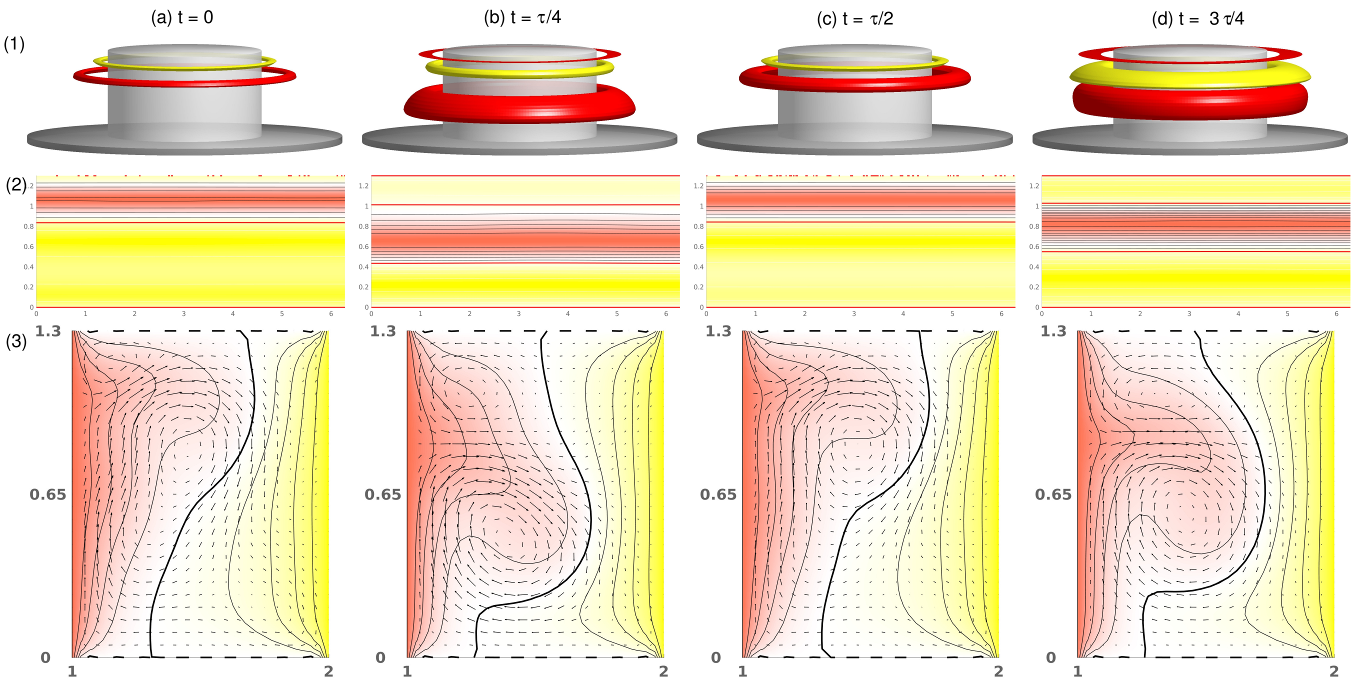

FIG. S2: **Visualization of the propagating flow state of pV$_2$, for $\alpha = 1$.** One period, τ, at instants of time, t, is shown as indicated: (1) isosurfaces of η (isolevel shown at $\eta = \pm 240$); (2) Azimuthal velocity at cylinder mid-height. Red (dark gray) and yellow (light gray) colors correspond to positive and negative values, respectively, with zero specified as white. (3) Vector plots $[u(r,z), w(r,z)]$ of the radial and axial velocity component, $(\theta = 0)$, where the color-coded azimuthal velocity field, v, is shown. The propagation period is $\tau_P \approx 0.896$. See also movie, movie_pV2_alpha1.avi.

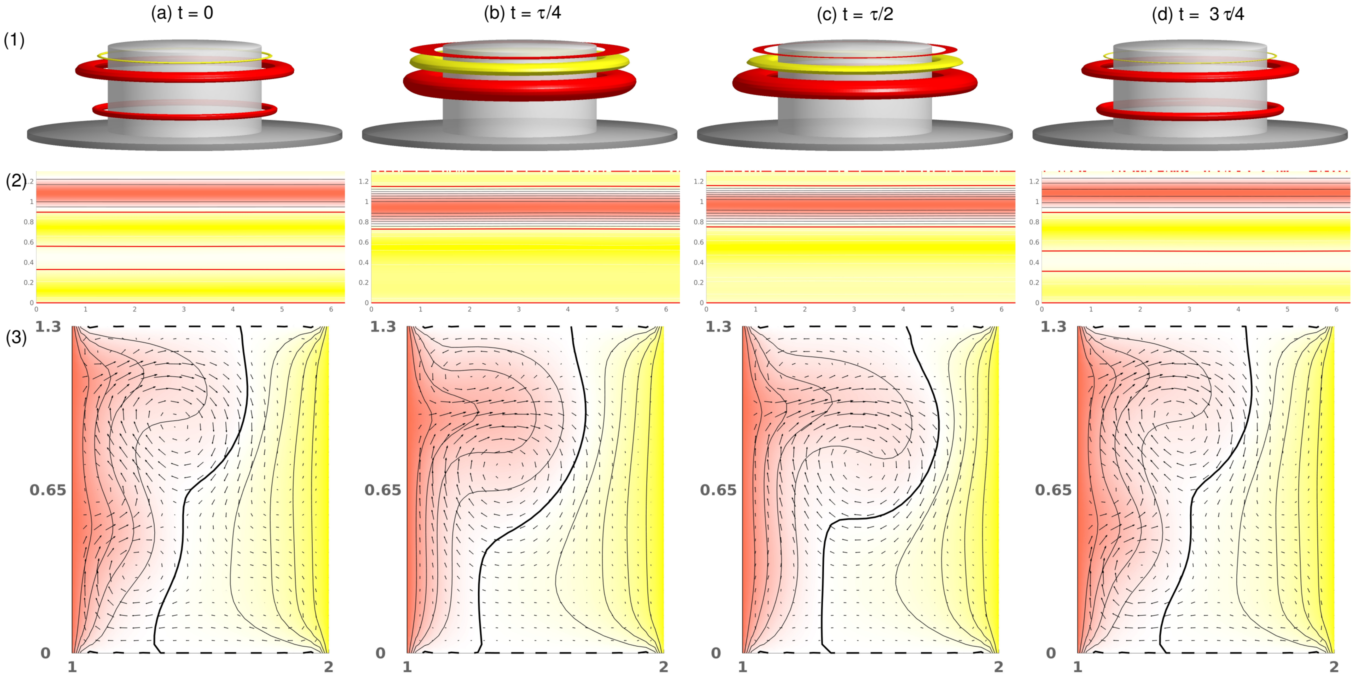

FIG. S3: **Visualization of the propagating flow state, pV$_2$, for** $\alpha = 0.7$**.** As shown in Fig. S2 with isosurfaces of $\eta = \pm 240$, the propagating period is $\tau_P \approx 1.395$. See also movie file movie_pV2_alpha07.avi in SMs.

Legends for videos in SM

- movie_T2_alpha-3_7.avi:
  movie_T2_alpha-3_7.avi demonstrates the T2$_2^{\text{puls}}$ solutions at $Re_1 = 250$, $Re_o = -250$, and $\alpha = -3.7$. Period time $\tau^{\text{puls}} \approx 0.194$. *Top left:* Radial velocity $u(\theta, z)$ on an unrolled cylindrical surface in the annulus at the mid-gap [red (yellow) color indicates out (in) flow]. *Top right:* Vector plots $[u(r,z), w(r,z)]$ of the radial and axial velocity component $(\theta = 0)$ where the color-coded azimuthal velocity field, v, is also shown. Solid black line indicates $v = 0$. *Bottom center:* Isosurfaces of the azimuthal vorticity, $\eta = \pm 240$ [red (yellow) color indicates positive (negative) vorticity]. *Bottom left:* Contours of azimuthal velocity field, v, in the (r, θ) plane at mid-height (viewed from the bottom). Thick black arrows highlight the rotation direction of the inner cylinder.

- movie_pV1_alpha0.avi:
  movie_pV1_alpha0.avi demonstrates the pV$_1$ solutions at $Re_1 = 250$, $Re_o = -250$, and $\alpha = 0$. Period time $\tau \approx 0.641$. *Top left:* Radial velocity $u(\theta, z)$ on an unrolled cylindrical surface in the annulus at the mid-gap [red (yellow) color indicates out (in) flow]. *Top right:* Vector plots $[u(r,z), w(r,z)]$ of the radial and axial velocity component $(\theta = 0)$, where the color-coded azimuthal velocity field, v, is also shown. Solid black line indicates $v = 0$. *Bottom center:* Isosurfaces of the azimuthal vorticity, $\eta = \pm 240$ [red (yellow) color indicates positive (negative) vorticity]. *Bottom left:* Contours of azimuthal velocity field, v, in the (r, θ) plane at mid-height (viewed from the bottom). Thick black arrows highlight the rotation direction of the inner cylinder.

- movie_pV2_alpha07.avi:
  movie_pV2_alpha07.avi demonstrates the pV$_2$ solutions at $Re_1 = 250$, $Re_o = -250$, and $\alpha = 0.7$. Period time $\tau \approx 1.395$. *Top left:* Radial velocity $u(\theta, z)$ on an unrolled cylindrical surface in the annulus at the mid-gap [red (yellow) color indicates out (in) flow]. *Top right:* Vector plots $[u(r,z), w(r,z)]$ of the radial and axial velocity component $(\theta = 0)$, where the color-coded azimuthal velocity field, v, is also shown. Solid black line indicates $v = 0$. *Bottom center:* Isosurfaces of the azimuthal vorticity, $\eta = \pm 240$ [red (yellow) color indicates positive (negative) vorticity]. *Bottom left:* Contours of azimuthal velocity field, v, in the (r, θ) plane at mid-height (viewed from the bottom). Thick black arrows highlight the rotation direction of the inner cylinder.

- movie_pV2_alpha1.avi:
  movie_pV2_alpha1.avi demonstrates the pV$_2$ solutions at $Re_1 = 250$, $Re_o = -250$, and $\alpha = 1$. Period time $\tau \approx 0.896$. *Top left:* Radial velocity $u(\theta, z)$ on an unrolled cylindrical surface in the annulus at mid-gap [red (yellow) color indicates

out (in) flow]. *Top right:* Vector plots $[u(r,z), w(r,z)]$ of the radial and axial velocity component $(\theta = 0)$, where the color-coded azimuthal velocity field, v, is also shown. Solid black line indicates $v = 0$. *Bottom center:* Isosurfaces of the azimuthal vorticity, $\eta = \pm 240$ [red (yellow) color indicates positive (negative) vorticity]. *Bottom left:* Contours of azimuthal velocity field, v, in the (r,θ) plane at mid-height (viewed from the bottom). Thick black arrows highlight the rotation direction of the inner cylinder.

- movie_pV2_alpha2.avi:
  movie_pV2_alpha2.avi demonstrates the pV_2 solutions at $Re_1 = 250$, $Re_o = -250$, and $\alpha = 2$. Period time $\tau \approx 0.405$. *Top left:* Radial velocity $u(\theta, z)$ on an unrolled cylindrical surface in the annulus at the mid-gap [red (yellow) color indicates out (in) flow]. *Top right:* Vector plots $[u(r,z), w(r,z)]$ of the radial and axial velocity component $(\theta = 0)$, where the color-coded azimuthal velocity field, v, is also shown. Solid black line indicates $v = 0$. *Bottom center:* Isosurfaces of the azimuthal vorticity $\eta = \pm 240$ [red (yellow) color indicates positive (negative) vorticity]. *Bottom left:* Contours of azimuthal velocity field, v, in the (r,θ) plane at mid-height (viewed from the bottom). Thick black arrows highlight the rotation direction of the inner cylinder.

---

[1] S. Hughes and A. Randriamampianina, *An improved projection scheme applied to pseudospectral methods for the incompressible Navier-Stokes equations* Int. J. Numer. Methods Fluids **28**, 501 (1998).

[2] I. Mercader, O. Batiste, and A. Alonso, *An efficient spectral code for incompressible flows in cylindrical geometries* Comput. Fluids **39**, 215 (2010).

[3] S. Altmeyer, C. Hoffmann, M. Heise, J. Abshagen, A. Pinter, M. Lücke, and G. Pfister, *End wall effects on the transitions between Taylor vortices and spiral vortices*. Phys. Rev. E **81**, 066313 (2010).

[4] J. M. Lopez and J. Shen, *An efficient spectral-projection method for the Navier-Stokes equations in cylindrical geometries I. Axisymmetric cases*, J. Comput. Phys. **139, 308-326 (1998).**
